# TIP60 enhances cisplatin resistance via regulating ΔNp63α acetylation in SCC

**DOI:** 10.1038/s41419-024-07265-6

**Published:** 2024-12-03

**Authors:** Akshay Hira, Jin Zhang, Madhavi P. Kadakia

**Affiliations:** Department of Biochemistry and Molecular Biology, Boonshoft School of Medicine, Wright State University, Dayton, OH USA

**Keywords:** Biochemistry, Cancer therapeutic resistance

## Abstract

Non-melanoma skin cancer, including basal and squamous cell carcinoma, is the most common form of cancer worldwide, with approximately 5.4 million new cases diagnosed each year in the United States. While the chemotherapeutic drug cisplatin is often used to treat squamous cell carcinoma (SCC) patients, low response rates and disease recurrence are common. In this study, we show that TIP60 and ΔNp63α levels correlate with cisplatin resistance in SCC cell lines, suggesting that TIP60 contributes to the failure of platinum-based drugs in SCC by regulating the stability and transcriptional activity of ΔNp63α. Depletion of endogenous TIP60 or pharmacological inhibition of TIP60 led to a decrease in ΔNp63α protein and acetylation levels in multiple SCC cell lines. We showed that TIP60 upregulates ΔNp63α protein levels in cisplatin-resistant SCC cell lines by protecting it from cisplatin-mediated degradation and increasing its protein stability. Stable expression of TIP60 or ΔNp63α individually promoted resistance to cisplatin and reduced cell death, while loss of either TIP60 or ΔNp63α induced G2/M arrest, increased cell death, and sensitized cells to cisplatin. Moreover, pharmacological inhibition of TIP60 reduced acetylation of ΔNp63α and sensitized resistant cells to cisplatin. Taken together, our study indicates that TIP60-mediated stabilization of ΔNp63α increases cisplatin resistance and provides critical insights into the mechanisms by which ΔNp63α confers cisplatin resistance by promoting cell proliferation and inhibiting apoptosis. Furthermore, our data suggests that inhibition of TIP60 may be therapeutically advantageous in overcoming cisplatin resistance in SCC and other epithelial cancers.

## Introduction

Squamous cell carcinoma (SCC) remains a pervasive form of skin cancer, exerting a substantial impact on global public health. In the United States alone, one million individuals are diagnosed with nonmelanoma skin cancer each year [1, 2]. Furthermore, Head and Neck SCC (HNSCC) contributes to over 800,000 new cases globally annually [3–5]. Cisplatin, a chemotherapeutic agent is frequently employed in the treatment of SCC across various origins [6]. Despite its effectiveness, more than 50% of advanced and non-resectable cutaneous SCC demonstrate resistance to this chemotherapeutic agent [7–9]. Therefore, understanding the mechanisms underlying cisplatin resistance is crucial for developing novel approaches to overcome drug resistance in the frontline treatment of SCC.

p63, a protein homologous to the well-known tumor suppressor protein p53, has been implicated as a master regulator of epidermal stratification and has a demonstrated role in maintaining the proliferative capacity of epithelial stem cells [10, 11]. ΔNp63α is the predominant p63 isoform in the basal layer of stratified epithelial tissues [12]. Elevated ΔNp63α levels are strongly associated with poor prognosis in SCC [13, 14]. ΔNp63α promotes tumor cell proliferation by inhibiting the transcription of cell cycle inhibitors such as p21, cyclin B2, and cdc2 [15–17]. Additionally, ΔNp63α has also been shown to negatively regulate the expression of apoptosis-related genes, thereby inhibiting cell death [18]. The transcriptional activity and stability of ΔNp63α are regulated by post-translational modifications (PTMs) [19, 20]. Lysine acetylation, mediated by lysine acetyltransferases (HATs), has been demonstrated to modulate the expression and activity of ΔNp63α [21, 22].

We previously identified the histone acetyltransferase (HAT) TIP60 as a novel key upstream regulator of ΔNp63α in SCC [22]. In this study, we have shown that high levels of ΔNp63α and TIP60 levels correlate with cisplatin resistance in SCC cell lines. We further report that TIP60 promotes cisplatin resistance by regulating ΔNp63α acetylation and protein stability in cisplatin-resistant cells. Our results clearly demonstrate that depletion of TIP60 or pharmacologic inhibition of TIP60 with NU9056 and TH1834 leads to a decrease in ΔNp63α acetylation, causes cell cycle arrest and promotes apoptotic cell death, thereby reducing cell survival in cisplatin-resistant cells. Together our results suggest that targeting the TIP60/ΔNp63α axis may sensitize resistant SCC cancer to cisplatin and points to the potential utility of TIP60 inhibition as an adjunct therapy to overcome cisplatin resistance, offering a promising new avenue for therapeutic interventions in non-melanoma skin cancer.

## Material and methods

### Cell lines, generation of stable cell lines, plasmids and reagents

A431 cells were purchased from American Type Culture Collection (Manassas, Virginia, USA) and maintained in Dulbecco’s modified Eagle’s medium (DMEM) supplemented with 10% fetal bovine serum (FBS) and 250 U penicillin and 250 µg streptomycin at 37 °C in 5% CO_2_. The cisplatin-resistant A431 variant cell line designated, A431 Pt and the corresponding parental cell lines were a generous gift from Dr. Paola Perego (Istituto Tumori di Milano, Milan, Italy) were established as described earlier [23]. A431 Parental and A431 Pt cells were cultured in RPMI 1640 medium supplemented with FBS and antibiotics as described above and maintained for no more than 20 passages to avoid morphological changes associated with extended culture. The naturally resistant Head and Neck SCC JHU006 and sensitive JHU029 cell lines obtained from Dr. James W Rocco (Ohio State University, Columbus, OH) were maintained in RPMI 1640 medium supplemented FBS and antibiotics as described above [24]. A431 and JHU029 cells stably expressing ΔNp63α (A431-ΔNp63α and JHU029-ΔNp63α), TIP60 (A431-TIP60 and JHU029-TIP60), or eGFP as a control (A431-eGFP and JHU029-eGFP) were generated by lentiviral-mediated transduction of A431 and JHU029 cells as described [22, 25]. At 72 h post infection, transduced cells were subjected to blasticidin antibiotic selection (3 µg/ml for A431 and 5 µg/ml for JHU029) (Life Technologies, Carlsbad, CA USA) to obtain cells stably expressing eGFP or ΔNp63α or TIP60. Cisplatin (cis-diammineplatinum (II) dichloride), purchased from Sigma-Aldrich (St. Louis, MO, USA), was used to prepare a 1 mg/ml cisplatin stock in 1× PBS. Cycloheximide, Carboplatin, Trichostatin-A, NU9056 and Nicotinamide were purchased from Sigma-Aldrich (St. Louis, MO). TH1834 was purchased from MedChemExpress (NJ, USA).

### Generation of stable doxycycline-inducible JHU006 cells expressing shRNA

JHU006 cells stably expressing doxycycline-inducible control shRNA (sh ctrl) or JHU006 shTIP60 (sh TIP60) were generated by lentiviral transduction. HEK-293FT cells were seeded onto a 10 cm dish for 24 h prior to transfection. Cells were transfected with lentivirus packaging plasmids 10 μg psPAX2, which express *Gag*, *Pol*, *Rev* and *Tat* genes and 6 μg pMD2.G, which expresses vesicular stomatitis virus G glycoprotein gene, along with 15 μg of scramble shRNA control or sh TIP60 cloned into the pTRIPZ lentiviral plasmid (Horizon Discovery, Waterbeach, Cambridge, UK). The scramble shRNA was obtained from Dr. Weiwen Long (Wright State University, Dayton, OH, USA). The shRNA targeting TIP60 was purchased from (Horizon Discovery, Waterbeach, Cambridge, UK). Cells were incubated for 5 h and media was changed to DMEM media with 8% FBS without antibiotics for virus production. At 72 h post transfection, media was collected, centrifuged to remove cell debris and filtered through a 0.25 μm filter. Virus containing media was incubated overnight with 2.5 ml of PEG-it virus precipitation solution. The following day, media was centrifuged, supernatant was removed and virus pellets were resuspended in 150 μl cold 1× PBS. JHU-006 cells were seeded in 6-well plates. The next day, 25 μl of lentivirus and 10 μg/ml of Polybrene (EMD Millipore, Billerica, MA, USA) were added to the JHU006 cells in 1 ml of complete RPMI media without antibiotics. At 48 h post transduction, the media was changed to fresh complete media containing puromycin antibiotic (2 μg/ml) (MP Biomedicals, Solon, OH, USA) to select for JHU006 cells stably expressing sh ctrl or shTIP60. The expression of scramble or TIP60 shRNA was induce by treating stable cell lines with 2 μg/ul Doxycycline for 4–5 days.

### siRNA transfections

AllStars negative control, non-silencing control (NSC), sip63 and siTIP60 siRNAs used in this study were purchased from Qiagen (Valencia, CA, USA) as described earlier [22]. Cells were transfected using Lipofectamine RNAi-Max (Life Technologies, Carlsbad, CA, USA) according to the manufacturer’s instructions, as reported previously [25, 26]. Cells were harvested 24 or 48 h after transfection. Resuspended cell pellets were used for immunoblotting.

### Immunoblot analysis

For immunoblotting, cells were lysed in buffer containing 50 mM Tris–HCl pH 8, 120 mM NaCl, 5 mM sodium pyrophosphate phosphatase inhibitor, 10 mM NaF, 30 mM paranitrophenylphosphate, 1 mM benzamidine, 0.1% NP-40, 1% Triton X-100, 0.2 mM PMSF, 100 nM sodium orthovanadate, and 10% protease inhibitor cocktail (Sigma, St. Louis, MO). Immunoblotting was carried out as previously described [25, 26]. Proteins were detected using rabbit polyclonal anti-GFP (FL) at 1:1000, anti-p63 (4A4) mouse monoclonal at 1:10,000, anti-TIP60 (C7, Santa Cruz Biotechnology, Santa Cruz, CA, USA) at 1:1000, anti-PARP, anti-Cleaved Casapase-3, p21(waf1/cip1) (Cell Signaling Technology, Danvers, MA, USA) at 1:1000, anti-gamma H2A.X (phospho S139) (Abcam, Cambridge, UK) at 1:10,000 and anti-β-actin mouse monoclonal (Santa Cruz Biotechnology, Santa Cruz, CA, USA) at 1:10,000. β-actin was used as a loading control. Horseradish peroxidase-conjugated secondary antibody (Promega, Madison, WI, USA) was used for chemiluminescence detection with the Western Lightning Plus kit (Perkin Elmer, Waltham, MA, USA) or SuperSignal West Femto Maximum Sensitivity Substrate (Thermo Fisher Scientific Inc., Waltham, MA, USA). Fold change in protein expression was calculated by normalizing band intensity to β-actin as a loading control using Multi Gauge software (Fujifilm, Tokyo, Japan).

### Immunoprecipitation assay for detection of acetylated proteins

Cells were treated with a combination of 1–2 μM Trichostatin A (HDAC class I and II inhibitor) and 5 mM Nicotinamide (HDAC class III inhibitor) for 6 h to inhibit the activity of HDACs and enrich for endogenous acetylated proteins. Cell lysates were prepared by sonicating cells in high salt lysis buffer (300 mM NaCl, 100 mM Tris, pH 8.0, 0.2 mM EDTA, 0.1% NP40 and 10% glycerol) supplemented with 1% protease inhibitor cocktail (Sigma, St. Louis, MO) and lysed on ice with intermittent vortexing. Protein concentrations were determined by BCA assay (Thermo Fisher Scientific Inc., Fremont, CA, USA). Equivalent protein amounts (1–2 mg) were pre-cleared with protein A agarose beads (Santa Cruz Biotechnology, Santa Cruz, CA, USA), followed by an overnight incubation with monoclonal anti-acetylated lysine antibody (Cell Signaling Technology, Danvers, MA, USA). The next day, protein-A beads were added, and the samples were rotated at 4 °C for 1 h, washed with PBS containing 0.05% Tween and analyzed by immunoblot analysis.

### Cell viability assay

For drug response, cells were seeded at 10,000 cells per well in a 96-well flat bottom culture dish for analysis of cell viability by MTS assay. At 24 h post plating, cells were pulsed for 2 h with cisplatin doses as indicated followed by complete medium lacking cisplatin. For measuring proliferation, cells were seeded in triplicates in a 96-well flat bottom culture dish at 2500 cells per well and cell proliferation was measured at 6, 24, 48 and 72 h after vehicle or drug treatment. Drug response or proliferation was measured by using the Cell Titer 96 AQueous One Solution Cell Proliferation Assay (MTS) (Promega, Madison, WI) performed according to the manufacturer’s instructions.

### Flow cytometry analysis

Cells transfected with the indicated siRNA were pulse treated with either 12.5 μg/ml or 42 μg/ml of cisplatin for 2 h followed by release in media without cisplatin. Cells were washed in PBS, fixed in 70% ethanol and incubated at −20 °C for 16 h. Fixed cells were then suspended in 500 μl of PBS containing 50 μg/ml propidium iodide and 100 μM RNase. Cells were then gated and analyzed using an Accuri C6 flow cytometer (BD Biosciences). Histograms of cell cycle phases were generated using FCS Express 4 (De Novo Software, Glendale, CA, USA).

### Quantitative RT-PCR

Total-RNA isolation from human cell lines was performed using the E.Z.N.A. Total RNA kit according to the manufacturer protocol (Omega Bio-Tek, Norcross, GA, USA). cDNA was synthesized from 1 µg of total RNA using the qScript cDNA Super Mix (Quantabio, Beverly, MA, USA). qRT-PCR was performed using the Applied Biosystem 7900HT or 7 Flex Real-Time PCR systems using Assays on Demand (AOD) specific for human β-globulin (Hs00187842_m1), BBC3 (Hs00248075_ml) and CDKN1A or p21 (Hs00355782_m1) (Life Technologies, Carlsbad City, CA, USA) with each sample run in technical triplicate. Relative expression was calculated using the ΔΔCT method with GAPDH used as an endogenous control as described previously [27, 28]. Significant differences (*p* ≤ 0.05) were identified using two-tailed Student’s t-tests assuming equal variances in samples compared to control.

### Statistical analysis

Independent-sample two-tailed *t-*tests for equal variance were performed to test for significant differences between experimental groups and controls when comparing two groups. Ordinary one-way ANOVAs were performed when comparing 3 or more groups with *p* values adjusted for multiple comparisons using Bonferroni correction. Differences were considered statistically significant at *p* ≤ 0.05.

## Results

### TIP60 and ΔNp63α are overexpressed in cisplatin-resistant cells

**Fig. 1:**
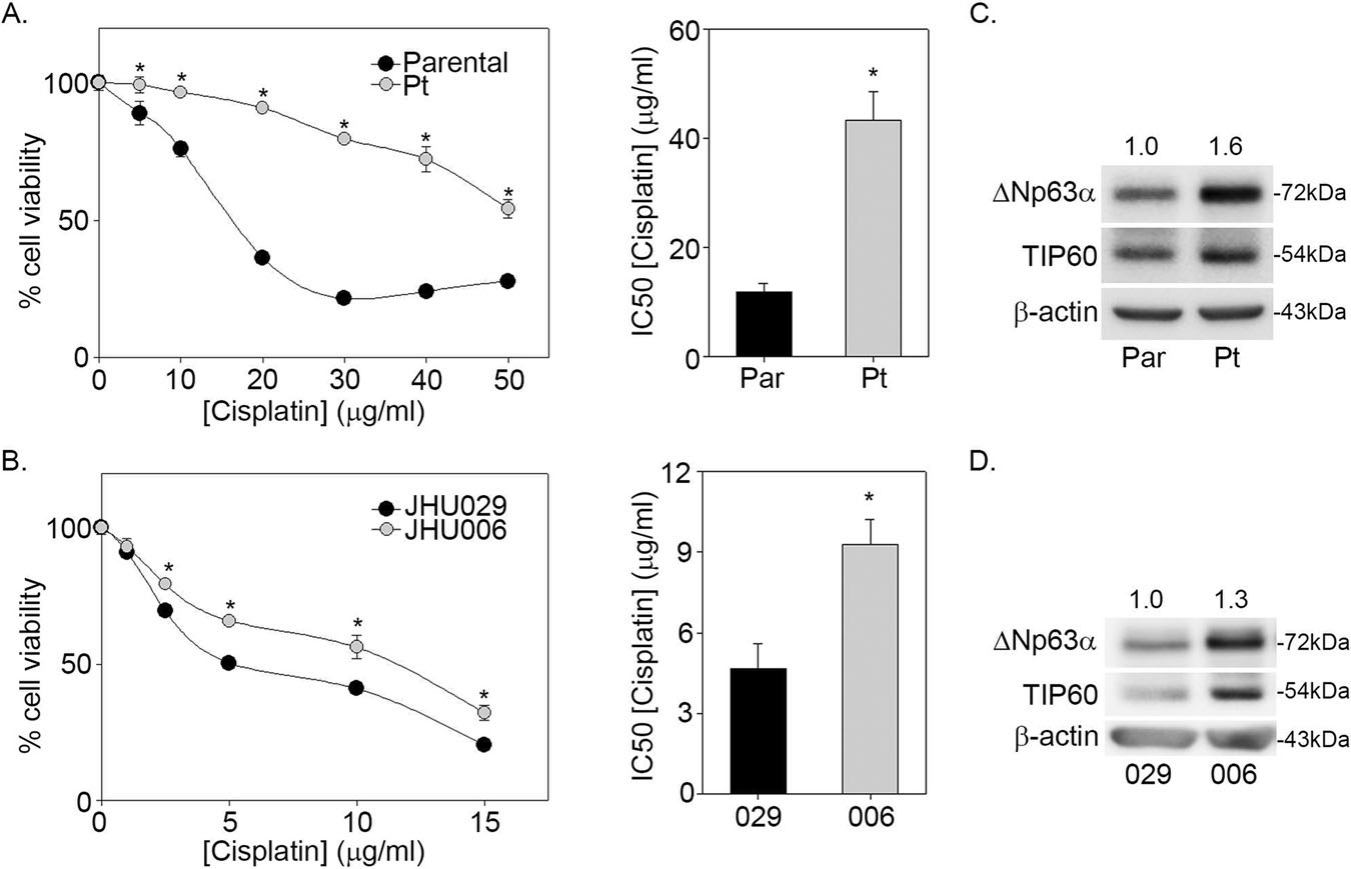
TIP60 and ΔNp63α are overexpressed in cisplatin-resistant cells. **A** A431 Parental and A431 Pt and **B** JHU029 and JHU006 cells were subjected to a 2-h cisplatin pulse treatment at the indicated doses. At 48-h post treatment, cell viability was measured by MTS assay (left panel). The *y*-axis indicates cell viability relative to vehicle-treated cells. The *x*-axis indicates the μg/μl concentration of cisplatin used for pulse treatment. Error bars represent ±1 SD from the mean. *p < 0.05 compared to respective control at each dose of cisplatin. Bar plots (right panel) show the mean IC_50_ value calculated from three independent experiments. Error bars indicate the mean +1 SEM from three independent experiments. *p < 0.05 com*p*ared to the IC50 value of A431 Parental or JHU029 sensitive controls. Immunoblot analysis performed on **C** A431 Parental and A431 Pt **D** JHU029 and JHU006 cells using antibodies specific for p63 and TIP60. Fold change in ΔNp63α protein relative to respective control is listed above each band. β-actin was included as a loading control. Representative blots are shown.

A431 Pt, A431 Parental, JHU006 and JHU029 cells were treated with increasing concentrations of cisplatin for measurement of cell viability. We observed a ~3.5-fold higher IC_50_ in cisplatin-resistant A431 Pt cells (IC_50_ = 43.2 ± 5.3 µg/ml) relative to cisplatin-sensitive A431 Parental cells (IC_50_ = 11.94 ± 1.5 µg/ml) (Fig. 1A), consistent as shown earlier [23]. JHU006 cells exhibit a ~2-fold higher IC_50_ (IC_50_ = 9.17 ± 0.5 µg/ml) relative to JHU029 cells (IC_50_ = 5.05 ± 0.6 µg/ml) (Fig. 1B). We observed a similar trend with carboplatin confirming the acquired resistant phenotype of A431 Pt cells to both the platinum-based drugs (Supplementary Fig. 1). To determine if ΔNp63α and TIP60 levels correlate with resistance to cisplatin, we next examined TIP60 and ΔNp63α levels in these cell line models. Immunoblot analysis showed higher expression of ΔNp63α and TIP60 in both A431 Pt and JHU006 cisplatin-resistant cell lines compared to the respective A431 Parental and JHU029 cisplatin-sensitive controls (Fig. 1C, D). These data indicate that TIP60 and ΔNp63α protein levels correlate with acquired and natural resistance to cisplatin in SCC cancer cells.

### Knockdown and pharmacological inhibition of TIP60 reduce ΔNp63α acetylation in cisplatin-resistant cells

**Fig. 2:**
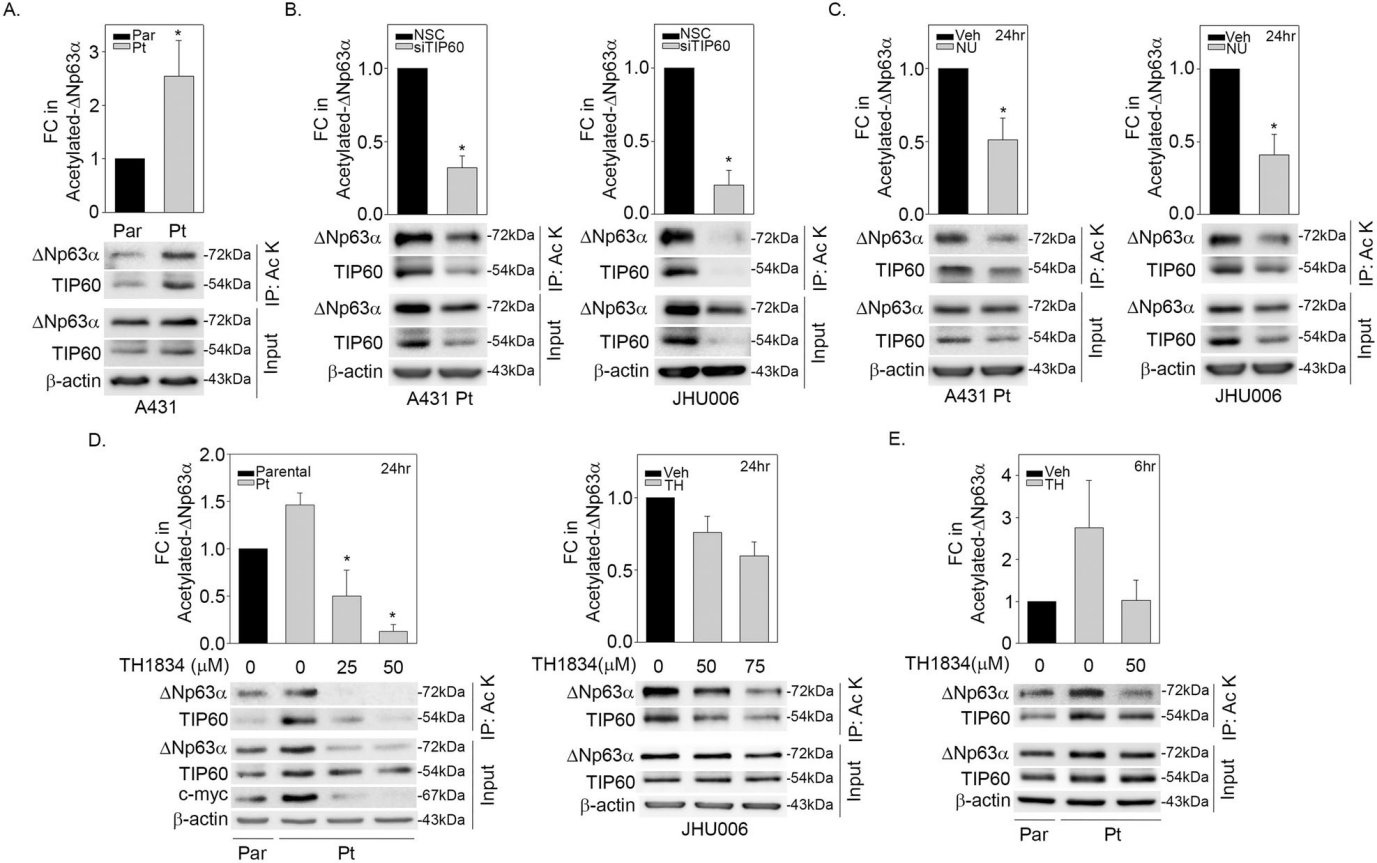
Knockdown and pharmacological inhibition of TIP60 reduces ΔNp63α acetylation in cisplatin-resistant cells. **A** A431 Parental and A431 Pt cells and **B** A431 Pt and JHU006 were transfected with non-silencing control (NSC) and si-RNA against TIP60 (siTIP60) as indicated. **C** A431 Pt and JHU006 cells were treated with either DMSO as a control (Veh, vehicle) or TIP60 inhibitor NU9056 at 100 μM and 86 μM dose, respectively. **D** A431 Parental and A431 Pt and JHU006 cells were treated with either DMSO as a control (Veh) or 25, 50 or 75 μM doses of TIP60 specific inhibitor TH1834 for 24 h as indicated. **E** A431 Parental and A431 Pt cells were transiently treated for 6 h with either DMSO as a control (Veh) or 50 μM dose of TIP60 specific inhibitor TH1834 as indicated and were harvested immediately. In all panels, cells were pre-treated with HDAC inhibitors 1 μM of Trichostatin A and 5 mM of Nicotinamide for 6 h prior to immunoprecipitation (IP). Whole-cell lysate cells were immunoprecipitated with an anti-acetyl-lysine (Ac-K) antibody followed by immunoblot analysis *(top panels)* using antibodies specific for p63, TIP60 or β-actin. β-actin was included as a loading control for equivalent protein in each IP and Input lane. Representative immunoblots are shown. Densitometric analysis *(top panel)* showing the fold change in acetylated-ΔNp63α relative to control condition after normalization to input β-actin. Error bars indicate mean +1SEM from three or more independent experiments. *p ≤ 0.05 relative to the corresponding Parental (**A**), NSC (**B**) or vehicle control (**C**, **D**).

We next sought to determine if ΔNp63α acetylation levels correlate with cisplatin resistance in SCC cell lines. We observed a positive correlation between higher TIP60 levels and increased ΔNp63α acetylation in cisplatin-resistant A431 Pt cells (Fig. 2A). It is well established that the auto-acetylation of TIP60 is critical for its enzymatic activity [29]. As expected, we also observed an increase in the acetylation of TIP60 in A431 Pt cells (Fig. 2A). Next, we determined whether TIP60 is required for elevated ΔNp63α acetylation in cisplatin-resistant cells by transiently transfecting A431 Pt cells and JHU006 cells with either non-silencing control (NSC) or siRNA against TIP60 (siTIP60). We observed a significant decrease in ΔNp63α acetylation upon silencing of endogenous TIP60 in both cisplatin-resistant cell lines models tested, confirming the effect is not cell line specific (Fig. 2B). To verify these findings, we investigated the effect of stable knockdown of TIP60 on endogenous ΔNp63α acetylation using TRIPZ doxycycline-inducible lentiviral shRNA TIP60 knockdown system. Stable knockdown of TIP60 led to a reduction in ΔNp63α acetylation in JHU006 shTIP60 cells compared to shctrl samples (Supplementary Fig. 2). Together, these results demonstrate that TIP60 is required for ΔNp63α acetylation in cisplatin-resistant cells.

To determine if ΔNp63α acetylation in cisplatin-resistant cell lines is dependent on TIP60 acetyltransferase activity, we assessed the effect of NU9056, an inhibitor of TIP60 activity, on ΔNp63α acetylation. A431 Pt and JHU006 cells were treated with either vehicle (DMSO) or with NU9056 at determined cell-line specific IC_50_ doses for 24 h (Supplementary Fig. 3). NU9056 decreased ΔNp63α acetylation in both cisplatin-resistant cell lines (Fig. 2C). Since NU9056 has been shown to exhibit off-target effects, we conducted parallel experiments using TH1834, an inhibitor of TIP60 acetyltransferase activity which has greater specificity than NU9056 [30]. Consistent with the effects of NU9056 treatment, TH1834 caused a dose-dependent reduction in ΔNp63α acetylation in both A431 Pt and JHU006 cells (Fig. 2D). To confirm that the observed decrease in ΔNp63α acetylation was not due to a decrease in ΔNp63α levels, A431 Pt cells were transiently treated with TH1834 for 6 h. TH1834 caused a reduction in ΔNp63α acetylation without decreasing ΔNp63α total levels in A431 Pt cells (Fig. 2E) indicating that upon TIP60 inhibition reduction in ΔNp63α acetylation precedes reduction in ΔNp63α protein levels. Taken together, these results indicate that TIP60 acetyltransferase activity is required for ΔNp63α acetylation in cisplatin-resistant cells.

### TIP60 protects ΔNp63α from cisplatin-mediated degradation

**Fig. 3:**
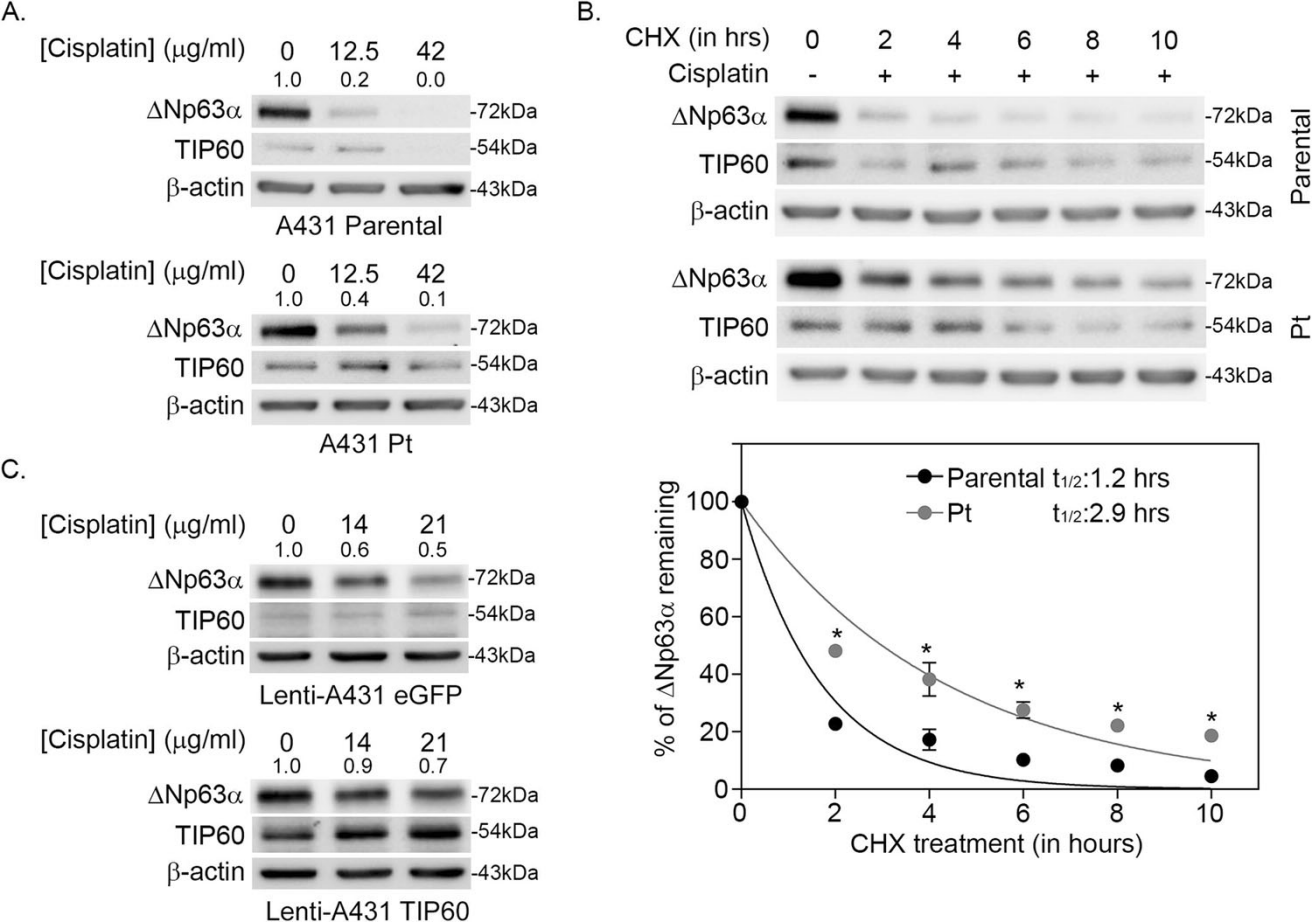
TIP60 protects ΔNp63α from cisplatin-mediated degradation. Immunoblot of **A** A431 Parental and A431 Pt cells treated either vehicle or cisplatin for 2-h pulse and harvested 24 h post pulse. **B** A431 Parental and A431 Pt cells were pulsed with cisplatin for 2 h and subsequently treated with cycloheximide 100 μg/ml cycloheximide (CHX) for 2, 4, 6, 8 and 10 h along with a non-treated control (0 h), respectively. The y-axis shows the percentage of ΔNp63α protein remaining relative to vehicle-treated cells at 0 h. ΔNp63α half-life (*t*_½_) was determined from the exponential curve equation calculated using the one-phase exponential decay model in GraphPad Prism 6 (bottom panel). Error bars indicate mean ± SEM from three independent experiments. *p ≤ 0.05 relative to Parental. **C** Lenti A431-eGFP and Lenti A431-TIP60 stable cells treated with vehicle or cisplatin for 2-h pulse and harvested 24 h later. Immunoblot analysis was performed using antibodies specific for p63, TIP60 or β-actin. Fold change in ΔNp63α protein relative to respective vehicle-treated control is listed above each band in (**A**, **C**). β-actin was included as a loading control for equivalent protein.

Previous reports have shown that the stability of ΔNp63α is regulated by post-translational modifications including phosphorylation and acetylation [31, 32]. We next sought to determine if cisplatin-resistant cells retained higher ΔNp63α levels than cisplatin-sensitive controls after cisplatin treatment. First, we compared the effect of cisplatin on ΔNp63α and TIP60 levels in A431 Parental and Pt cells. Pt cells retained higher levels of ΔNp63α and TIP60 relative to Parental cells in response to cisplatin treatment (Fig. 3A).

TIP60 maintains the stability of various non-histone proteins in a manner dependent on its catalytic activity [33–37]. Therefore, we investigated whether the elevated levels of ΔNp63α exhibited increased protein stability in cisplatin-resistant cells. Cisplatin-resistant A431 Pt cells exhibited an increased ΔNp63α protein half-life (*t*_1/2_ = 2.9 h) relative to Parental controls (*t*_1/2_ = 1.2 h) upon exposure to cisplatin (Fig. 3B).

Next, to determine if TIP60 protects ΔNp63α from cisplatin-mediated degradation, Lenti-A431 eGFP and Lenti-A431-TIP60 stable cell lines were treated with either vehicle (PBS) or the IC_50_ doses for Lenti-A431-eGFP (IC_50_ = 14 µg/ml) or Lenti-A431-TIP60 (IC_50_ = 21 µg/ml). Lenti-A431 TIP60 with stable TIP60 expression showed increased ΔNp63α levels following cisplatin treatment relative to Lenti-A431 eGFP controls cells (Fig. 3C) indicating that TIP60 protects ΔNp63α from cisplatin-mediated degradation. These results together suggest that ΔNp63α has an increased half-life and stability in cisplatin-resistant cell lines.

### TIP60 promotes ΔNp63α protein stability in cisplatin-resistant cells

**Fig. 4:**
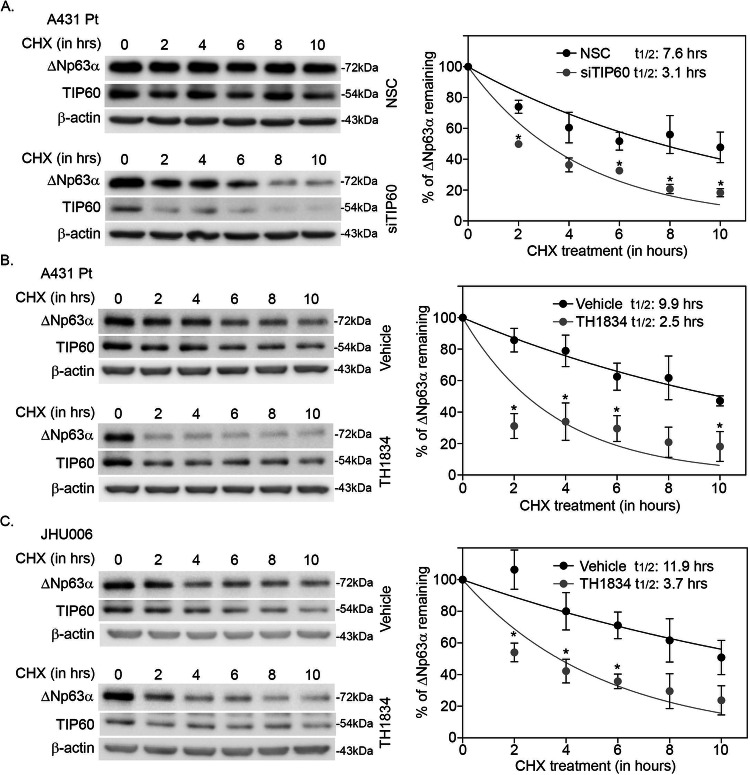
TIP60 promotes ΔNp63α protein stability in cisplatin-resistant cells. **A** A431 Pt cells were transfected with non-silencing control (NSC) and si-RNA against TIP60 (siTIP60) as indicated. **B** A431 Pt and **C** JHU006 cells were pre-treated with either DMSO (Veh, vehicle control) or 50 μM TH1834 for 16 h. **A**–**C** Cells were treated with cycloheximide 100 μg/ml cycloheximide (CHX) for 2, 4, 6, 8 and 10 h along with a non-treated control (0 h), respectively. Immunoblot analysis (left panels) was performed using antibodies specific for p63, TIP60 or β-actin. β-actin was included as a loading control for equivalent protein. The y-axis (right panels) shows the percentage of ΔNp63α protein remaining relative to untreated cells at 0 h. ΔNp63α half-life (*t*_½_) was determined from the exponential curve equation calculated using the one-phase exponential decay model in GraphPad Prism 6. Error bars indicate mean ± SEM from three independent experiments. *p ≤ 0.05 relative to **A** NSC or **B**, **C** vehicle-treated cells.

To investigate whether the observed increase in ΔNp63α stability in cisplatin-resistant cells is dependent on TIP60, A431 Pt cells were transfected with non-silencing control (NSC) or si-RNA against TIP60 (siTIP60) and treated with cycloheximide to block de novo protein synthesis. TIP60 knockdown reduced the half-life of ΔNp63α (*t*_1/2_ = 3.1 h) when compared to non-silencing controls (*t*_1/2_ = 7.6 h) (Fig. 4A), suggesting that TIP60 protects ΔNp63α from cisplatin-mediated degradation in cisplatin-resistant cells. To confirm that TIP60 catalytic activity is required for the increased ΔNp63α protein stability observed in cisplatin-resistant cells, we tested the effects of the TIP60-specific inhibitor TH1834 on ΔNp63α protein half-life in A431 Pt and JHU006 cells. TIP60 inhibition decreased ΔNp63α protein stability both in A431 Pt (*t*_1/2_ = 2.5 h) and JHU006 (*t*_1/2_ = 3.7 h) (Fig. 4B, C) relative to vehicle-treated A431 Pt (*t*_1/2_ = 9.9 h) and JHU006 (*t*_1/2_ = 11.9 h) controls. These data demonstrate that TIP60 promotes ΔNp63α stability in cisplatin-resistant cells in a manner dependent on TIP60 catalytic activity. Taken together, these results suggest that TIP60 both acetylates ΔNp63α and promotes its stability in the presence and absence of cisplatin.

### Knockdown or pharmacological inhibition of TIP60 decreases ΔNp63α and sensitizes cells to cisplatin

**Fig. 5:**
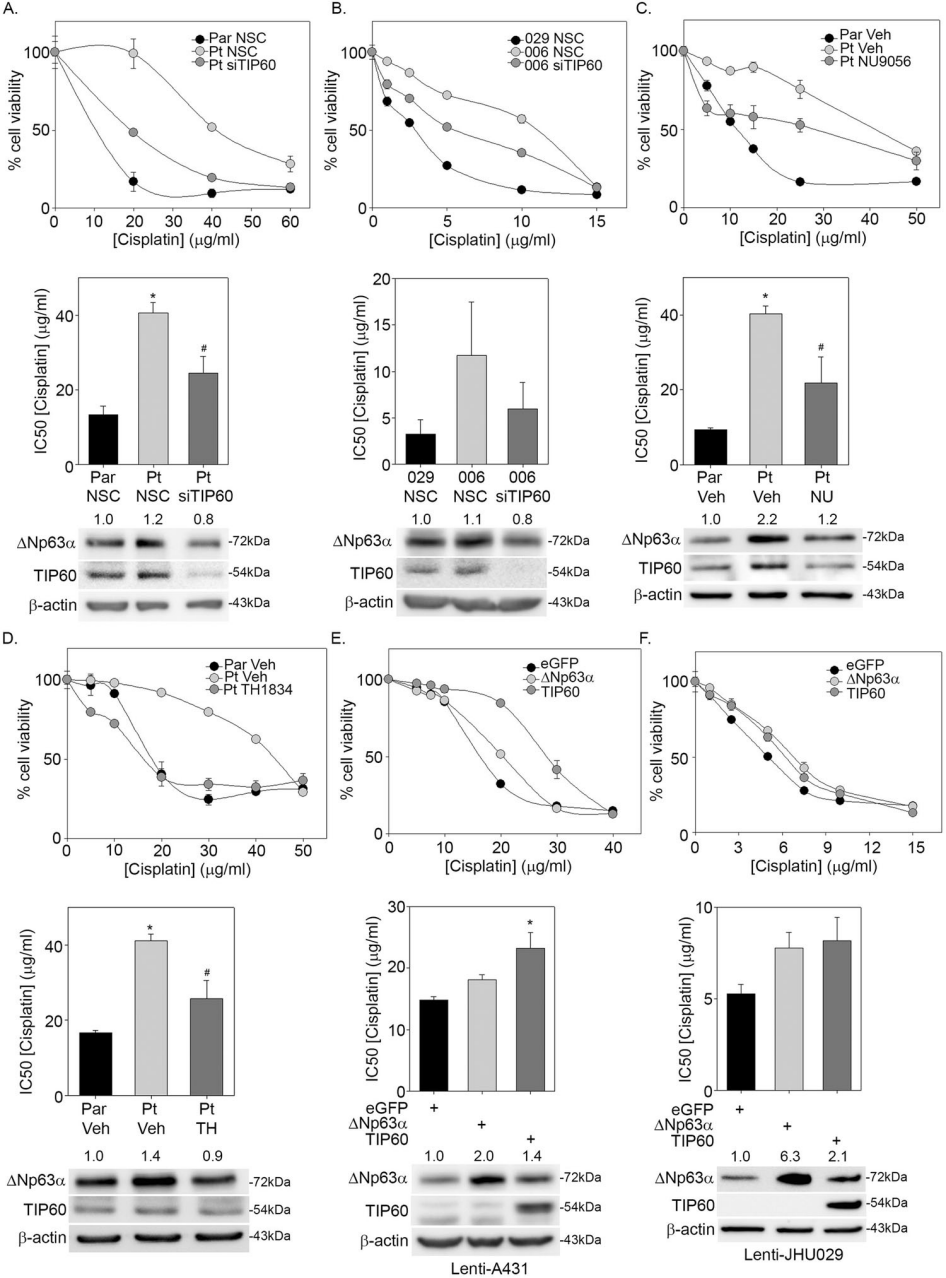
Knockdown or pharmacological inhibition of TIP60 decreases ΔNp63α and sensitizes cells to cisplatin. **A** A431 Parental and A431 Pt **B** JHU029 and JHU006 were transfected with non-silencing control (NSC) siRNA or siRNA against TIP60 (siTIP60) and subjected to a 2-h cisplatin pulse treatment at the indicated doses. MTS was performed at 48-h post treatment. A431 Parental (control) and **C** Pt cells were treated with either vehicle or 100 μM NU9056, and **D** Pt cells were pre-treated with either vehicle or 75 μM TIP60 specific inhibitor TH1834 for 6 h. Cells were then subjected to a 2-h cisplatin pulse at the indicated doses and MTS was performed at 48-h post treatment. *p ≤ 0.05 compared to A431 Parental vehicle control and ^#^*p* ≤ 0.05 compared to respective A431 Pt vehicle control at each dose of cisplatin. **E** Lenti A431-eGFP (control), ΔNp63α and TIP60 **F** Lenti JHU029-eGFP (control), ΔNp63α and TIP60 cells were subjected to a 2-h cisplatin pulse treatment at the indicated doses and MTS was performed at 24-h post treatment. *p < 0.05 compared to the Lenti-eGFP controls. Cell viability (y-axis) from an experiment representative of three independent experiments is shown in the top panels in **A**–**F**. The *x*-axis indicates the μg/μl concentration of cisplatin used for pulse treatment. Error bars indicate mean ±1SD from three technical replicates. Bar plots (middle panels in **A**–**F**) show the mean IC_50_ + 1 SEM values calculated from three independent experiments. Immunoblot analysis (bottom panels in **A**–**F**) performed using antibodies specific for p63 and TIP60 is shown. Fold change in ΔNp63α protein relative to respective NSC or vehicle-treated control is listed above each band. β-actin was included as a loading control for equivalent protein.

Since ΔNp63α has been linked to cisplatin resistance in ovarian and lung cancer [38, 39], we next examined whether TIP60-mediated cisplatin resistance in SCC cells is ΔNp63α dependent. A431 Parental cells showed the expected dose dependent decrease in viability in response to cisplatin treatment (IC50 = 13.3 µg/ml) (Fig. 5A). A431 Pt cells transfected with NSC showed the expected increase in cisplatin resistance (IC50 = 40.5 µg/ml) relative to A431 Parental control. Interestingly, TIP60 knockdown significantly sensitized A431 Pt cells to cisplatin (IC50 = 24.4 µg/ml), although not quite to the level observed in A431 Parental cells (Fig. 5A). Similarly, TIP60 knockdown in JHU006 cells which exhibit a higher baseline cisplatin resistance effectively sensitized cells to cisplatin (Fig. 5B).

The TIP60 small-molecule inhibitor NU9056 has been shown to decrease the stability of TIP60 by blocking its autoacetylation, resulting in reduced cell proliferation [40]. NU9056 treatment significantly sensitized A431 Pt cells to cisplatin (Fig. 5C, *top and middle panel*). NU9056 treatment of A431 Pt cells also decreased ΔNp63α protein levels, as expected (Fig. 5C, *bottom panel*). Similar to the effects of NU9056 treatment, the TIP60-specific inhibitor TH1834 treatment similarly sensitized A431 Pt cells to cisplatin (Fig. 5D, *top and middle panel*). These results together indicate TIP60 inhibition sensitizes chemo-resistant SCC cells to cisplatin and reduces ΔNp63α levels. These data suggest that TIP60 and ΔNp63α may limit the effectiveness of cisplatin in resistant cells and suggest a potential therapeutic utility of TIP60 inhibitors in cisplatin-resistant cancers.

To determine if stable expression of ΔNp63α and TIP60 could increase resistance to cisplatin, we generated A431 and JHU029 stable cell lines by lentiviral-mediated transduction of ΔNp63α, TIP60 or eGFP (control). Stable expression of ΔNp63α (A431-ΔNp63α and JHU029-ΔNp63α) increased resistance to cisplatin relative to controls (A431-eGFP and JHU029-eGFP) (Fig. 5E, F), (A431-eGFP IC50 = 14.8 and A431-ΔNp63α IC50 = 18.1 μg/ml, and JHU029-eGFP IC50 = 5.3 and JHU029-ΔNp63α IC50 = 7.8 μg/ml). Interestingly, stable expression of TIP60 (A431-TIP60 and JHU029-TIP60), like ΔNp63α, resulted in a significant increase in cisplatin resistance compared to A431-eGFP and JHU029-eGFP controls (Fig. 5E, F), (A431-eGFP IC50 = 14.8 and A431-TIP60 IC50 = 23.3 μg/ml and JHU029-eGFP IC50 = 5.3 and JHU029-TIP60 IC50 = 8.2 μg/ml). Together these results indicate that TIP60 promotes cisplatin resistance by increasing ΔNp63α protein levels. Further, since stable expression of TIP60 showed the highest resistance to cisplatin, this data suggests that TIP60 promotes resistance in a manner dependent on ΔNp63α.

### Pharmacological inhibition of TIP60 decreases proliferation in cisplatin-resistant cells

**Fig. 6:**
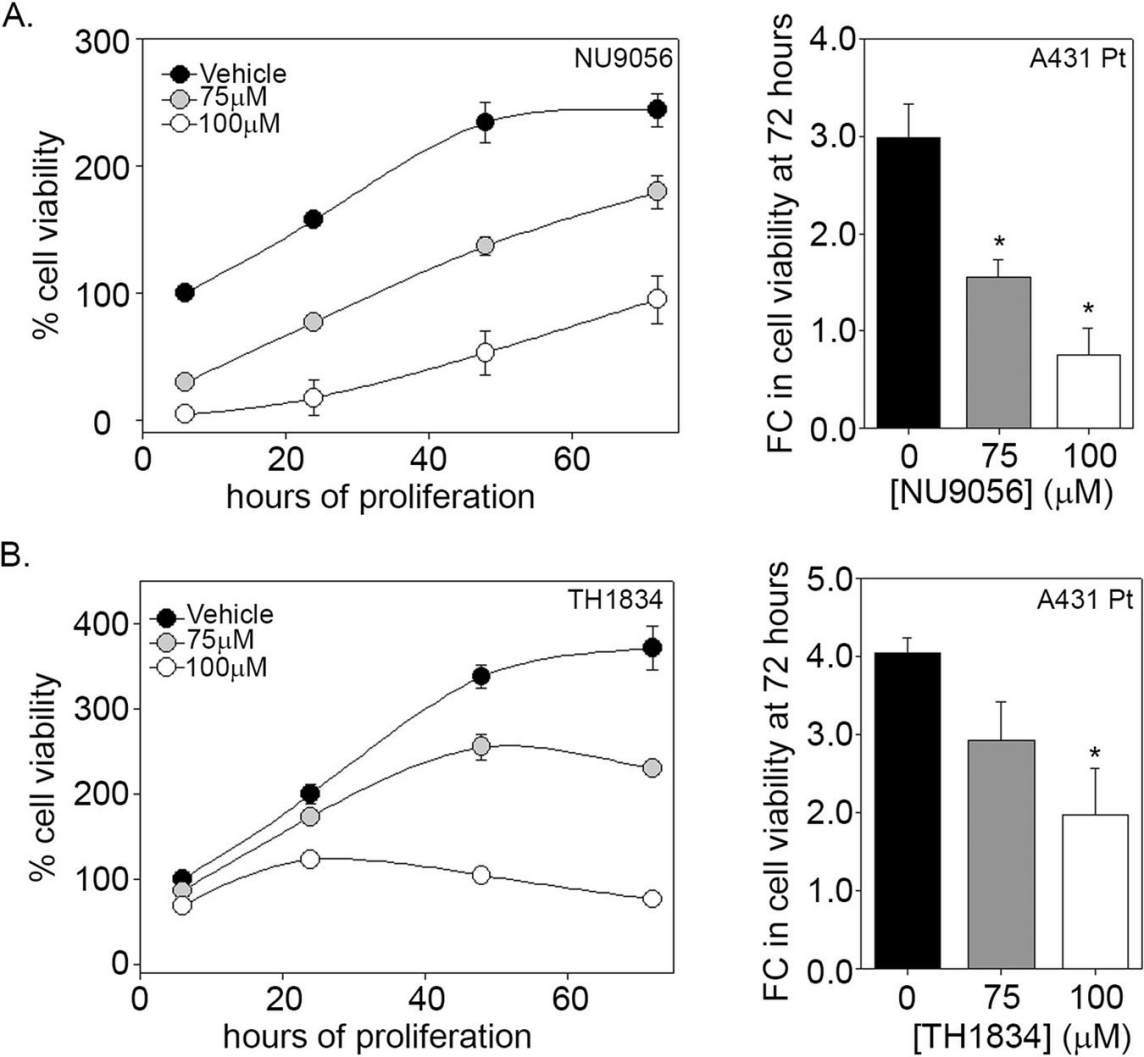
Pharmacological inhibition of TIP60 decreases proliferation in cisplatin-resistant cells. Cell viability was measured by MTS analysis (*left panels*) at 6, 24, 48 and 72 h as indicated on the x-axis. A431 Pt cells were treated with either vehicle (DMSO) or **A** 75 μM or 100 μM NU9056, **B** 75 μM and 100 μM TH1834 as indicated. The *y*-axis indicates viability relative to vehicle-treated cells at 6 h. Error bars represent ± 1SD from the mean from samples measured in technical triplicate. Bar-plot (right panels) shows the fold change in viability at 72-h relative to the 6-h vehicle control. *p ≤ 0.05 compared to vehicle-treated condition. Error bars indicate the mean + 1SEM from three independent experiments.

Previous studies indicate that TIP60 promotes cell proliferation in various cancer types, including colon cancer, lung cancer, colorectal cancer, and prostate cancer [41–43]. Similarly, ΔNp63α is well-established for its oncogenic properties and its role in promoting cancer cell proliferation [13, 44, 45]. Therefore, we investigated the effect of TIP60 pharmacological inhibition of TIP60 on cell proliferation in cisplatin-resistant A431 Pt cells. NU9056 caused a dose-dependent decrease in cell proliferation compared to the vehicle-treated control Pt cells (Fig. 6A), with significant reductions at all doses at 72 h post treatment (Fig. 6A, right panel). Similar to NU9056, TH1834 treatment caused a dose-dependent decrease in proliferation in cisplatin-resistant cells compared to the vehicle-treated control (Fig. 6B) with significant reductions in proliferation evident at 72 h post treatment (Fig. 6B, right panel). These findings underscore the critical role of TIP60 acetyltransferase activity in promoting proliferation in cisplatin-resistant cells.

To determine if TIP60 promotes proliferation in a manner dependent on ΔNp63α, we performed rescue experiment using cell lines generated by lentiviral-mediated transduction of A431 Parental and JHU029 cells, which stably express ΔNp63α (A431-ΔNp63α and JHU029-ΔNp63α) or enhanced green fluorescent protein (A431-eGFP or JHU029-eGFP), the latter serving as a control. We observed that stable expression of ΔNp63α led to an increase in proliferation compared to the control A431 and JHU029 cells expressing eGFP (Supplementary Fig. 4A, D, E). Importantly, while TIP60 silencing decreased proliferation in both cell lines when exposed to cisplatin, stable expression of ΔNp63α partially rescued the loss of cell proliferation caused by TIP60 knockdown (Supplementary Fig. 4A, Veh and cis) and conferred increased resistance to cisplatin to levels comparable to those observed in A431-eGFP NSC cells (Supplementary Fig. 4B). TH1834 treatment decreased proliferation in both cell lines, while stable expression of ΔNp63α partially rescued the loss of cell proliferation caused by TIP60 inhibition in Lenti-A431 and JHU029 cells expressing ΔNp63α (Supplementary Fig. 4D, E). These results together indicate that TIP60 promotes ΔNp63α dependent cell proliferation in cisplatin-resistant cells and thereby plays an important role in the regulation of cellular response to cisplatin.

### TIP60 and ΔNp63α inhibit cell-cycle arrest and cell death in cisplatin-resistant cells

**Fig. 7:**
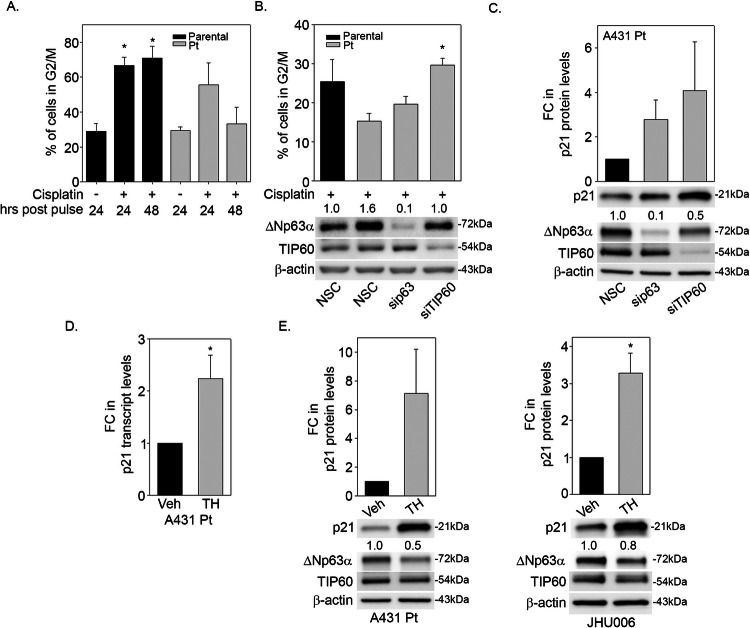
TIP60 and ΔNp63α inhibit cell-cycle arrest in cisplatin-resistant cells. **A** Percentage of cells progressing through G2/M at 24 and 48-h post 2-h cisplatin pulse treatment in A431 Parental and Pt cells measured by flow cytometry using FCS Express 4. The *y*-axis indicates % of cells in G2/M cell cycle phase. *p ≤ 0.05 compared to vehicle-treated condition for the corresponding cell line at 24 h post pulse. **B** A431 Parental and Pt cells were transfected with NSC, sip63 or siTIP60. Cells were pulsed with 12.5 μg/ml cisplatin for 2-h and subjected to flow analysis 48 h later. Cell cycle profiles were analyzed using Flow Cytometry. The *y*-axis indicates the percentage of cells in G2/M cell-cycle phase. *p ≤ 0.05 compared to Pt (NSC). Representative blot is shown. **C** A431 Pt cells were transfected with NSC, sip63 and siTIP60 and harvested at 48 h post transfection. **D** A431 Pt cells were treated with either Vehicle (DMSO) or 50 μM of TH1834, TIP60 specific inhibitor as indicated. At 24 h post treatment, cells were harvested, and total RNA was extracted, and p21 transcript levels were measured by TaqMan-based qRT-PCR. The y-axis indicates the fold change in transcript levels relative to the control. **E** A431 Pt and JHU006 cells were treated with either Vehicle (DMSO) or 50 μM of TH1834, TIP60 specific inhibitor as indicated. Changes in p21, ΔNp63α and TIP60 protein levels were measured by immunoblot analysis performed using antibodies specific for p21, p63, TIP60 or β-actin, as indicated. Fold change in ΔNp63α protein relative to respective NSC or vehicle-treated control is listed above each band in (**B**, **C**, **E**). β-actin was included as a loading control for equivalent protein. Densitometric analysis (top-panel) showing the fold change in p21 levels relative to Pt (Veh) and JHU006 (Veh) and normalized to β-actin. Error bars indicate means +1SEM from two (**A**) to three (**B**–**E**) independent experiments. *p ≤ 0.05 relative to Pt (NSC) as control.

ΔNp63α and TIP60 are known to regulate several genes involved in cell-cycle regulation [22, 46]. To determine if TIP60 and ΔNp63α contribute to cisplatin resistance by inhibiting cell-cycle arrest and cell death, we first investigated the effects of cisplatin on G2/M arrest in A431 Parental and A431 Pt cells. Both A431 Parental and A431 Pt cells showed an accumulation of cells in G2/M 24-h after cisplatin pulse (Fig. 7A). A further accumulation of A431 Parental cells was observed in G2/M by 48 h, consistent with cisplatin-induced cell cycle arrest. By contrast, the fraction of A431 Pt cells in G2/M reduced by 48 h, indicating progression through the cell cycle and a reduced level of G2/M arrest compared to A431 Parental cells (Fig. 7A). These results suggest that cisplatin-resistant cells with higher ΔNp63α and TIP60 levels have an increased capacity to bypass cisplatin-induced G2/M arrest than cisplatin sensitive cells.

Knockdown of either ΔNp63α or TIP60 in cisplatin-resistant cells increased the proportion of A431 Pt cells in G2/M compared to NSC controls (Fig. 7B), indicating that ΔNp63α and TIP60 promote cell cycle progression in cisplatin-resistant cells. We also showed that knockdown of ΔNp63α or TIP60 increased p21 (waf1/cip1) protein levels in cisplatin-resistant cells (Fig. 7C). Furthermore, TIP60 inhibition also increased p21 transcript levels in A431 Pt cells (Fig. 7D). Similar to the effects of TIP60 knockdown, TH1834 treatment increased p21 (waf1/cip1) protein levels in both A431 Pt and JHU006 resistant cells (Fig. 7E).

**Fig. 8:**
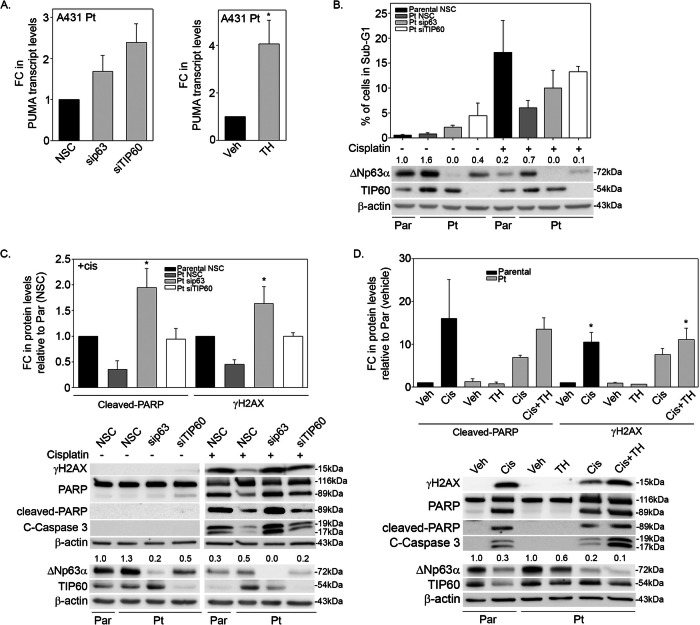
TIP60 and ΔNp63α inhibit cell death and apoptosis in cisplatin-resistant cells. **A** A431 Pt cells *(left-panel)* transfected with NSC, sip63 and siTIP60 and harvested at 48 h post transfection. A431 Pt cells (right-panel) were treated with either Vehicle (DMSO) or 50 μM of TH1834 as indicated. Cells were harvested, total RNA extracted, and PUMA transcript levels were measured by TaqMan-based qRT-PCR. The y-axis indicates the fold change in transcript levels relative to the corresponding NSC or vehicle (Veh) control. Error bars indicate means + 1SEM from three independent experiments. **B** A431 Parental and Pt cells were transfected with NSC, sip63 or siTIP60 and analyzed by flow cytometry at 48 h post transfection. The y-axis indicates the percentage of cells in Sub-G1 cell-cycle phase. Error bars indicate the mean + 1SEM from three independent experiments. Immunoblot analysis (bottom panel) was performed to measure the changes in ΔNp63α and TIP60 protein levels and confirm silencing. Immunoblot analysis was performed using antibodies specific for p63, TIP60 or β-actin. β-actin was included as a loading control for equivalent protein. Representative blot is shown. **C** A431 Parental and Pt cells were transfected with NSC, sip63 and siTIP60. Cells were pulsed with vehicle or 42 μg/ml of cisplatin for 2-h and were subjected to immunoblot analysis 48 h later. **D** A431 Parental and Pt cells pulse treated with vehicle or 42 μg/ml of cisplatin for 2-h followed by continuous treatment with either vehicle or TH1834 (50 μM) for 24 h. Immunoblot was performed using antibodies specific for γ-H2AX, PARP, cleaved PARP, cleaved caspase-3, p63, TIP60 or β-actin. Fold change in ΔNp63α protein relative to respective NSC or vehicle-treated control is listed above each band in (**B**, **C**, **D**). β-actin was included as a loading control for equivalent protein in each lane. Densitometric analysis *(top panels)* showing the fold change in cleaved-PARP and γ-H2AX levels normalized to β-actin. Error bars indicate as mean + 1SEM from three independent experiments. * *p* ≤ 0.05 relative to **A** vehicle-treated Pt cells and **C** Parental NSC (cisplatin-treated) **D** vehicle-treated Parental or Pt cells as control. Representative blot is shown.

ΔNp63α has been shown to negatively regulate the transcription of apoptosis-related genes, thereby inhibiting cell death [18]. Knockdown of either ΔNp63α or TIP60 in cisplatin-resistant cells increased the transcript levels of PUMA in A431 Pt cells compared to NSC controls, indicating that depletion of ΔNp63α and TIP60 promotes cell death via apoptosis (Fig. 8A, *left panel*). Similarly, inhibition of TH1834 also increased PUMA transcript levels in A431 Pt cells (Fig. 8A, right panel). Since we observed knockdown of ΔNp63α or TIP60 both reduce cell proliferation by promoting G2/M arrest (Fig. 7B), we next sought to determine whether silencing of ΔNp63α and TIP60 increased cisplatin-induced cell death by measuring the percent of cells in sub-G1, a readout of cell death and DNA fragmentation [47]. The percent of cells in sub-G1, was determined using flow cytometry [47]. In vehicle-treated cells, knockdown of ΔNp63α and TIP60 resulted in an increased fraction of cells in the sub-G1 phase compared to the NSC control. Cisplatin treatment caused an increase in cell death in A431 Parental cells relative to vehicle controls. Knockdown of ΔNp63α and TIP60 in A431 Pt cells increased the percentage of cells in sub-G1 phase indicating increased cell death in response to cisplatin (Fig. 8B). Taken together, these results suggest that ΔNp63α and TIP60 contribute to cisplatin resistance by promoting cell cycle progression and inhibiting cell death.

Cisplatin is well known to induce cell death by promoting apoptosis [18, 48]. We next examined whether knockdown of either ΔNp63α or TIP60 promotes apoptosis-mediated cell death in cisplatin-resistant cells. In the absence of cisplatin, silencing TIP60 increased cleavage of PARP, a substrate of caspase in cisplatin-resistant cells (Fig. 8C). Upon cisplatin treatment, we observed an increase in cleaved-PARP and cleaved-Caspase-3 levels in A431 Parental cells, while NSC control cisplatin-resistant A431 Pt cells showed a reduced level of cell death markers, as expected. Knockdown of ΔNp63α or TIP60 in cisplatin-treated A431 Pt cells both increased the expression of cell death and apoptotic markers (Fig. 8C), although the effect of TIP60 knockdown on cleaved PARP did not reach statistical significance. H2AX, a histone variant of H2A, gets phosphorylated upon cytotoxic and genotoxic stress, resulting in activation of DNA damage signaling [49, 50]. Phosphorylation of H2AX at serine position 139 is considered a novel marker for DNA damage and double-strand breaks [51, 52]. As expected, we observed no phosphorylation of H2AX protein in the vehicle-treated cells. Parental cells showed increased γH2AX levels upon exposure to cisplatin compared to Pt cells. Importantly, knockdown of ΔNp63α and TIP60 in cisplatin-treated A431 Pt cells increased phosphorylation of H2AX (Fig. 8C), although the effect of TIP60 knockdown on cleaved PARP did not reach statistical significance. Taken together, this data indicates an increase in cisplatin-induced DNA damage in response to ΔNp63α and TIP60 silencing.

In parallel, we used a pharmacological approach to determine the combined effect of TIP60 inhibition and cisplatin on DNA damage and apoptosis in cisplatin-resistant cells. The combination of TH1834 and cisplatin treatment resulted in an increase in the levels of apoptotic markers relative to either treatment alone, suggesting TH1834 enhances the pro-apoptotic and cell death-promoting activity of cisplatin in cisplatin-resistant cells (Fig. 8D). TH1834 treatment alone did not induce γH2AX. Interestingly, the combined treatment of cisplatin and TH1834 increased γH2AX levels in Pt cells (Fig. 8D). Taken together, these results indicate that depletion of either TIP60 or ΔNp63α enhances the ability of cisplatin to induce cell cycle arrest resulting in apoptotic cell death in cisplatin-resistant cells.

## Discussion

The development of drug resistance is a major obstacle in the treatment of cancer. In this study, we investigated the role of TIP60 and ΔNp63α in acquired and natural resistance to cisplatin. We showed that cells with natural and acquired cisplatin resistance express higher levels of TIP60 and ΔNp63α compared to their cisplatin-sensitive counterparts. Moreover, we observed cross-resistance to carboplatin in the acquired cisplatin-resistant cell line.

TIP60 is a histone acetyltransferase that has been shown to regulate DNA damage response and repair [53–55]. Previous studies have reported that TIP60 expression is upregulated in cisplatin-resistant lung cancer cells and that TIP60 knockdown sensitizes cells to cisplatin-induced apoptosis [56]. Similarly, upregulation of ΔNp63α in head and neck cancer cells has been shown to confer resistance to cisplatin, while knockdown of ΔNp63α sensitizes these cells to cisplatin-induced apoptosis [38, 48]. Our findings are consistent with these studies and suggest that TIP60 and ΔNp63α play a role in cisplatin resistance SCC.

TIP60 has been shown to acetylate and enhance the protein stability of several transcription factors including c-MYC and p53 [33–37]. Our findings demonstrate that TIP60 promotes chemoresistance in SCC by acetylating ΔNp63α and increasing ΔNp63α levels and stability. Importantly, loss of ΔNp63α acetylation showed a resulting reduction in total ΔNp63α levels, consistent with our published finding that acetylation by TIP60 stabilizes ΔNp63α protein [22]. NU9056, while considered a TIP60 inhibitor, also targets other histone acetyltransferase (HAT) enzymes such as p300, PCAF, and GCN5 [40]. Based on our observation that TIP60 levels decreased in response to NU9056 treatment, it appears likely that off-target inhibition of p300 or other histone acetyltransferases (HATs) may have contributed to the reduction in TIP60 total levels with NU9056. By contrast, although treatment with the TIP60-specific TH1834 inhibitor resulted in the expected decrease in both ΔNp63α acetylation and total levels in cisplatin-resistant cell lines, TH1834 decreased TIP60 acetylation without effecting total TIP60 levels. This finding suggests that TH1834 does not exhibit the same off-target effects as observed with NU9056 (e.g. p300 inhibition), consistent with prior reports that TH1834 has higher specificity for TIP60 [57]. Thus, our findings underscore the reported specificity of TH1834 for TIP60 inhibition and suggest it would have fewer off-target effects than alternatives like NU9056 in the treatment of cisplatin-resistant squamous cell carcinoma (SCC).

ΔNp63α is frequently degraded by ubiquitin-proteasome-dependent pathways [58–60]. Several E3 ligases have been identified as key regulators of ΔNp63α protein levels, including MDM2, Pirh2, ITCH/AIP4, NEDD4, WWP1, and CHIP [61–67]. These E3 ligases play crucial roles in ubiquitinating ΔNp63α, thereby marking it for degradation by the proteasome. Furthermore, phosphorylation of ΔNp63α promotes its degradation in response to DNA damage [32, 68]. Cisplatin induces the ATM-dependent phosphorylation of ΔNp63α resulting in an increase in proteasomal degradation and a resulting reduction in ΔNp63α protein levels [32, 68]. ΔNp63α undergoes phosphorylation and subsequent translocation to the cytoplasm in response to cisplatin, where it becomes targeted for proteasome-mediated degradation by the protein RACK1 [20]. Similar reductions in ΔNp63α levels have also been observed following treatment with UV and paclitaxel [19]. However, the mechanism by which ΔNp63α acetylation promotes cisplatin resistance and ΔNp63α stability had not been explored earlier. Further investigation is required to test whether acetylation of ΔNp63α blocks phosphorylation and ultimately ubiquitination to prevent ΔNp63α degradation. The identification and characterization of lysine acetylation sites on ΔNp63α will provide valuable insights into the molecular mechanisms underlying its functional regulation.

ΔNp63α has been associated with increased expression of genes implicated in proliferation and cell cycle progression [13, 44, 45, 69–71]. Similarly, overexpression of TIP60 has been shown to enhance cell proliferation by promoting lysine acetylation of the androgen receptor (AR) [72, 73]. Consistent with previous observations, we observed a dose-dependent decrease in proliferation of cisplatin-resistant upon silencing or pharmacological inhibition of TIP60. These findings suggest that TIP60 activity is critical for maintaining the proliferative capacity of cisplatin-resistant cells.

Cisplatin is well-known for its capacity to induce a G2/M phase cell cycle arrest, serving as a protective mechanism against DNA damage [74]. As expected, our findings revealed that cisplatin-resistant cells exhibited the capability to bypass the G2/M arrest induced by cisplatin, unlike their parental counterparts. We also showed that knockdown of TIP60 and ΔNp63α resulted in an increase in G2/M arrest and p21 transcript and protein levels in untreated and cisplatin-treated Pt cells. Taken together, these findings suggest the significance of ΔNp63α and TIP60 levels in the promotion of cell cycle progression in response to cisplatin. ΔNp63α also controls the expression of apoptosis-related genes through transcription, thereby hindering cell death [18]. While inhibition of TIP60 has been shown to promote apoptosis in prostate cancer [40], the role of TIP60 in apoptosis in SCC has not been explored. Our findings reveal that knockdown of TIP60 and ΔNp63α increased the percentage of cells in the Sub-G1 phase of the cell cycle and increased the expression of apoptotic gene PUMA, indicating increased cell death and apoptosis. These findings suggest that TIP60 and ΔNp63α may limit the cytotoxic effects of cisplatin by inhibiting cell death in response to cisplatin. TH1834 treatment has been shown to promote the activation of apoptotic pathways in breast cancer cells [57]. These findings suggest a combined effect of TH1834 and cisplatin in inducing cell death in resistant cells. These data provide compelling support indicating a novel role for TIP60 in promoting chemoresistance by both reducing apoptotic cell death and promoting cell survival.

Unlike p63, increased activation of p53 and p73 in response to cisplatin has been associated with the promotion of apoptosis and cell death [75, 76]. The ΔNp63α transcription factor has structural similarities with other members of the p53 family, including p73, making direct therapeutic targeting of ΔNp63α challenging [77–79]. Our findings and prior reports highlighting TIP60’s potential role in chemoresistance development [22], suggest that inhibiting TIP60 may be a viable approach to reduce ΔNp63α levels and sensitize cisplatin-resistant tumors to chemotherapy. Further, whether TIP60 modulates chemoresistance through its regulation of p53 or p73 proteins remains unclear and an area of future study.

Histone acetyltransferases (HATs) remain attractive targets for cancer therapy despite known challenges related to target specificity [80]. Recent advances in targeting HAT enzyme activity or complex formation have led to the identification of inhibitors with improved efficacy [81], although comprehensive toxicity data remain limited. Naturally occurring acetyltransferase inhibitors such as garcinol [82] and curcumin exhibit minimal toxicity, suggesting that the development of safe inhibitors is feasible. TIP60 inhibitors such as NU9056 and TH1834 have proven useful tools in studying HAT function, but additional research is required to assess their in vivo efficacy and toxicity. Both inhibitors have shown antitumor efficacy in animal models [83–86], but formal toxicity studies are still lacking. Further preclinical research using patient-derived xenograft (PDX) SCC models is necessary to gather critical in vivo toxicity data and to evaluate the efficacy of TIP60 inhibitors in reducing tumor growth and sensitizing tumors to cisplatin, advancing this promising therapeutic strategy.

In conclusion, the findings presented in this study reveal a novel regulatory mechanism by which TIP60 and ΔNp63α contribute to cisplatin resistance in squamous cell carcinoma cell lines. The data shows that TIP60 promotes the expression and protein stability of ΔNp63α in cisplatin-resistant cells, resulting in reduced cell cycle arrest, apoptotic cell death and DNA damage in response to cisplatin. This important finding suggests that the combination of TIP60 inhibitors with platinum-based chemotherapy could elicit a better clinical response in therapy-resistant SCC cancers.

## Supplementary information

Supplemental Figures with Legends_no mark up

Supplemental material Westrn blot images
