# Supplementary material for: TIP60 enhances cisplatin resistance via regulating ΔNp63α acetylation in SCC
Source: Cell Death Dis. 2024 Dec 3;15(12):877. doi: 10.1038/s41419-024-07265-6 (PMC11615348; doi:10.1038/s41419-024-07265-6)
Supplement: Supplementary file 1 — Supplemental Figures with Legends_no mark up [file 41419_2024_7265_MOESM1_ESM.docx]

**
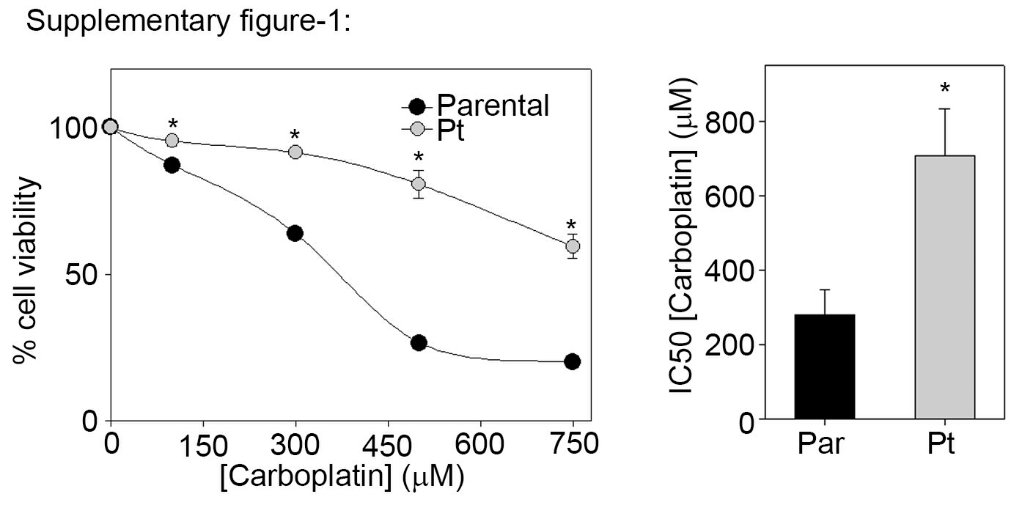
**

**Supplementary figure-1:** **Cisplatin-resistant A431 Pt cells show resistance to carboplatin.** A431 Parental (control) and A431 Pt (acquired cisplatin-resistant) cells were subjected to carboplatin treatment at the indicated doses. At 48-hour post-treatment, cell viability was measured by MTS assay. The y-axis indicates cell viability relative to vehicle treated cells. The x-axis indicates the μM concentration of carboplatin used for treatment. Error bars represent ±1 standard deviation from the mean of technical triplicates. **p*<0.05 compared to respective control at each dose of carboplatin. Bar plots *(right panel)* show the mean IC_50_ value calculated from three independent experiments. Error bars indicate means +1 S.E.M from three independent experiments. **p*<0.05 compared to the IC_50_ value of A431 Parental sensitive controls.

**
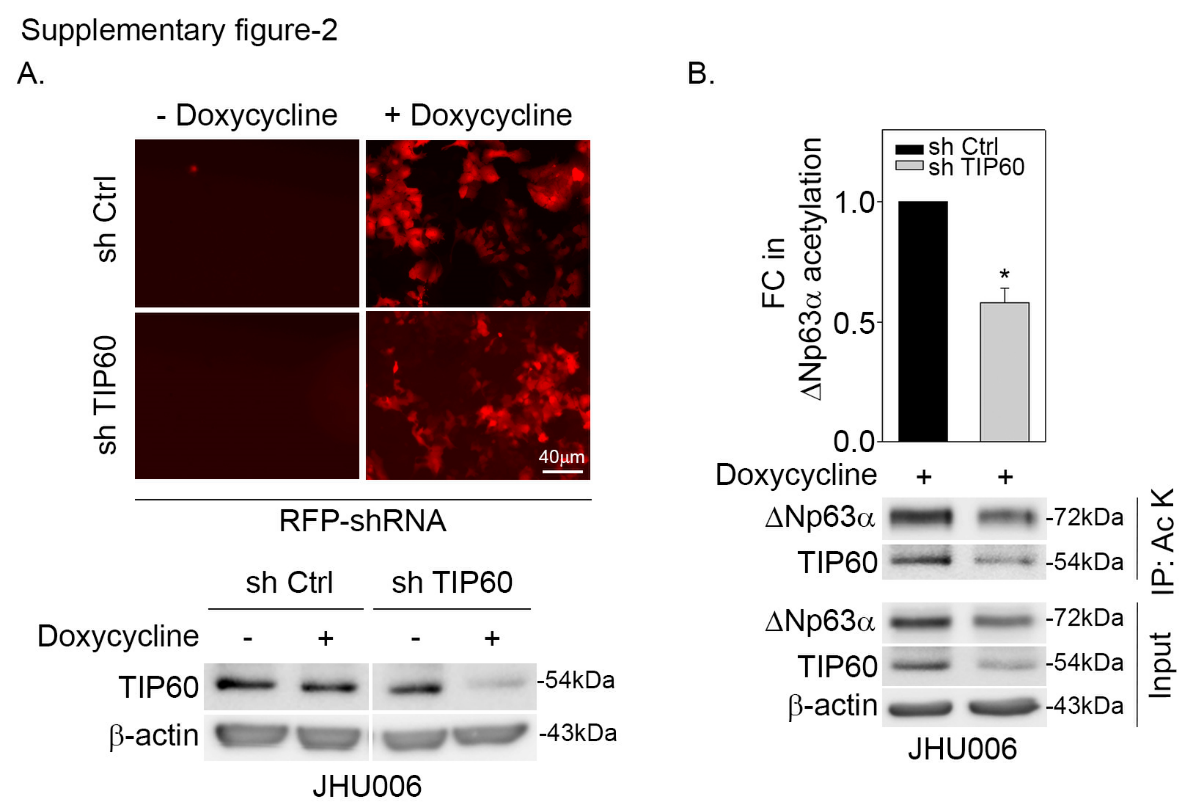
**

**Supplementary figure-2: Stable knockdown of TIP60 reduces ΔNp63α acetylation in cisplatin-resistant cells.** (A) JHU006 cells were transduced with lentivirus expressing scrambled shRNA as a control (sh Ctrl) or with shRNA targeting TIP60 (sh TIP60) and treated with Puromycin for selection of stable clones. Post-transduction, cells were treated with 2μg/ml of Doxycycline for 120 hours to induce shRNA expression. Red-fluorescence protein (RFP) induction was monitored microscopically, and images were taken using a 40x objective. Bottom panel shows TIP60 expression determined by immunoblot analysis using antibodies specific for TIP60 or β-actin. (B) Whole cell lysates from JHU006 sh ctrl and sh TIP60 stable cells were immunoprecipitated with an anti-acetyl-lysine (Ac-K) antibody followed by immunoblot analysis using antibodies specific for p63, TIP60 or β-actin. β-actin was included as a loading control for equivalent protein in each IP and Input lane. Densitometric analysis (top panel) showing the fold change in acetylated-ΔNp63α relative to shCtrl after normalization to input β-actin. Error bars indicate mean ± S.E.M. from four independent experiments. * *p* ≤ 0.05 relative to the shCtrl cell line.

**
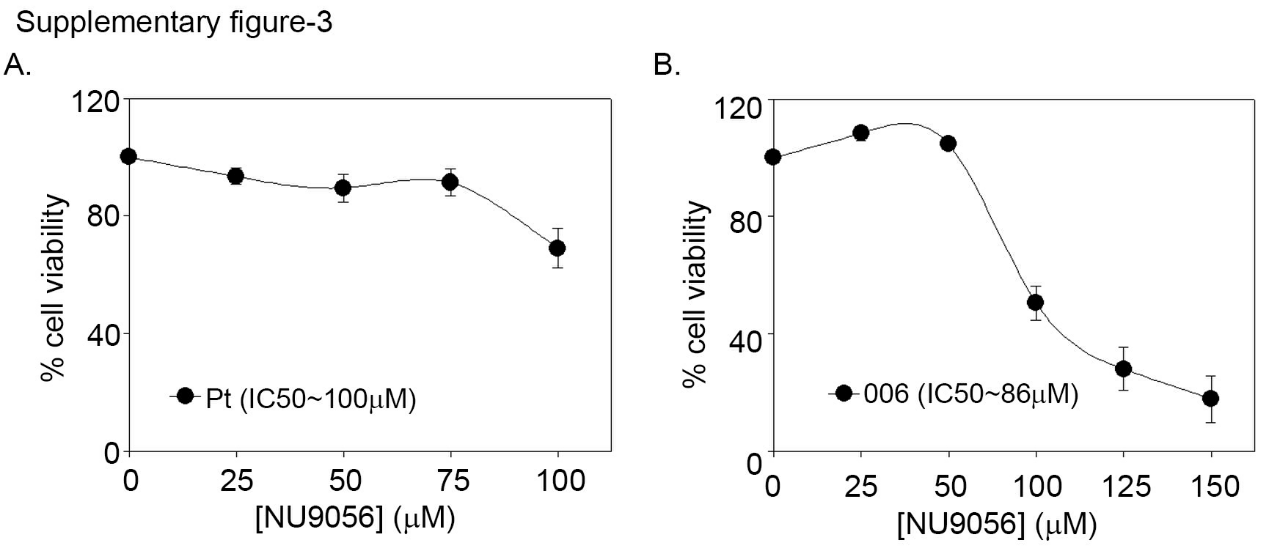
**

**Supplementary figure-3:** **Dose curve for NU9056 using cisplatin resistant cell lines** (A) A431 Pt and (B) JHU006 cells. Cells were treated with vehicle (0μM) and increasing concentrations of NU9056. At 24-hour post-treatment, cell viability was measured by MTS assay. The y-axis indicates cell viability relative to vehicle treated cells. Error bars indicate means +1 S.E.M from three independent experiments. The x-axis indicates the μΜ concentration of NU9056 treatment. Legend inset lists the calculated IC_50_ value.

**
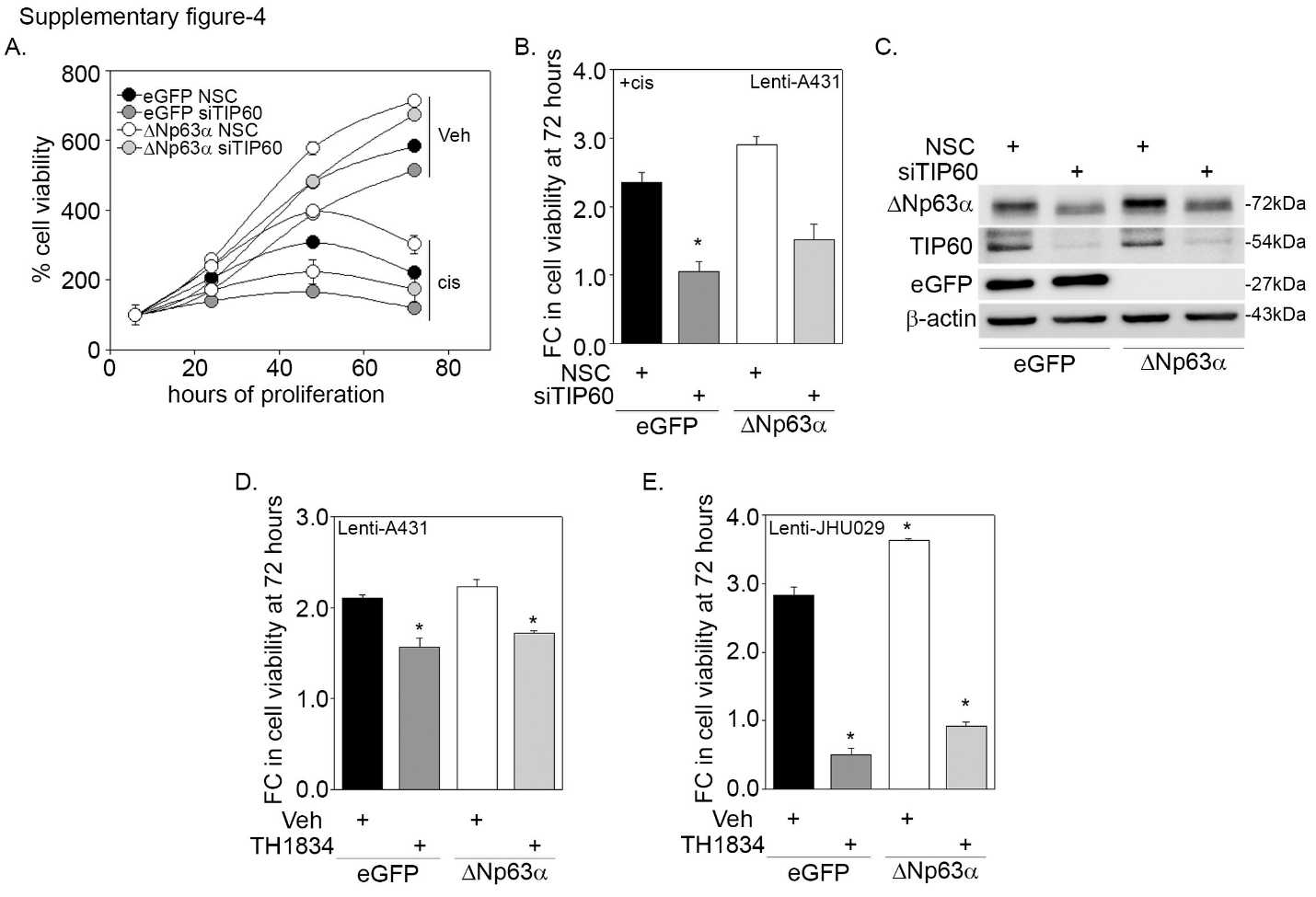
Supplementary figure-4: ΔNp63α partially restores cisplatin resistance and proliferation following TIP60 knockdown and inhibition.** (A) Cell viability was measured by MTS analysis of Lenti A431-eGFP (control) and A431-ΔNp63α stable cells transfected with non-silencing control siRNA (NSC) or siRNA against TIP60 (siTIP60). Cells were subjected to a 2-hour cisplatin pulse at 5μg/ml dose and MTS was performed at 6, 24, 48 and 72 hours post-treatment. Time-course showing viability relative to vehicle-treated cells at 6 hours is shown on the y-axis. (B) Bar-plot showing the fold change in viability at 72-hour time point. **p*≤0.05 relative to the corresponding A431-eGFP NSC control normalized to the NSC condition at 6-hours. Error bars indicate means +1 S.E.M from two independent experiments. (C) Immunoblot analysis was performed to measure the changes in ΔNp63α and TIP60 protein levels upon silencing of TIP60. Immunoblot analysis was performed using antibodies specific for p63, TIP60 or β-actin. β-actin was included as a loading control for equivalent protein. Representative blot shown. Cell viability was measured by MTS analysis of (D) Lenti A431-eGFP (control) and A431-ΔNp63α stable cells (E) Lenti JHU029-eGFP (control) and JHU029-ΔNp63α stable cells treated with vehicle (Veh) or 50μM of TH1834. Bar-plot showing the fold change in viability at 72-hour time point. **p*≤0.05 relative to the corresponding A431-eGFP NSC control normalized to the NSC condition at 6-hours. Error bars from three technical replicates represent +1 standard deviation from the mean.
