# Supplementary material for: TIP60 enhances cisplatin resistance via regulating ΔNp63α acetylation in SCC
Source: Cell Death Dis. 2024 Dec 3;15(12):877. doi: 10.1038/s41419-024-07265-6 (PMC11615348; doi:10.1038/s41419-024-07265-6)

Supplementary material: Western blot images

Figure-1C (A431 Parental and Pt)

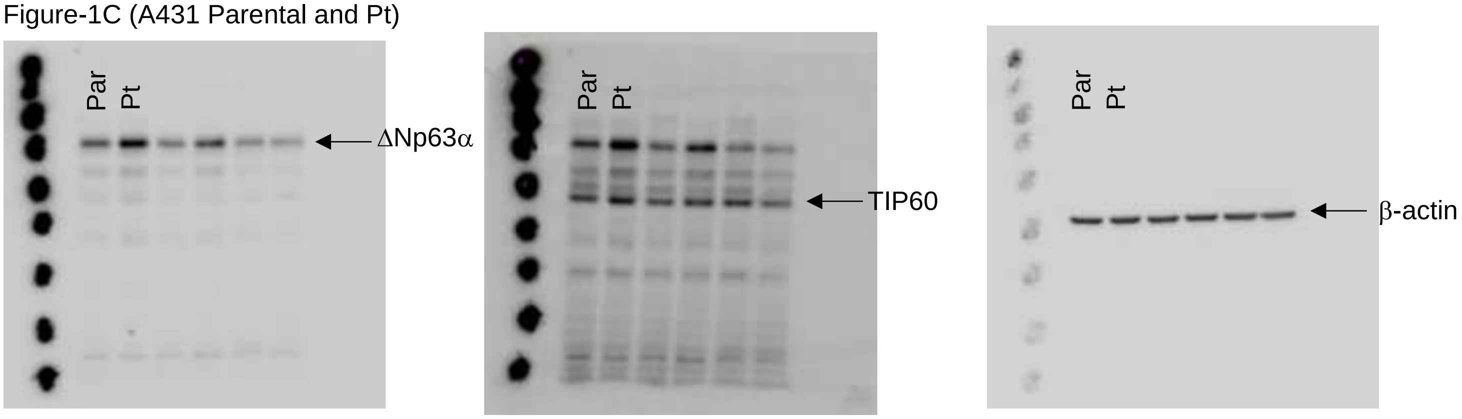

Figure-1D (JHU029 and JHU006)

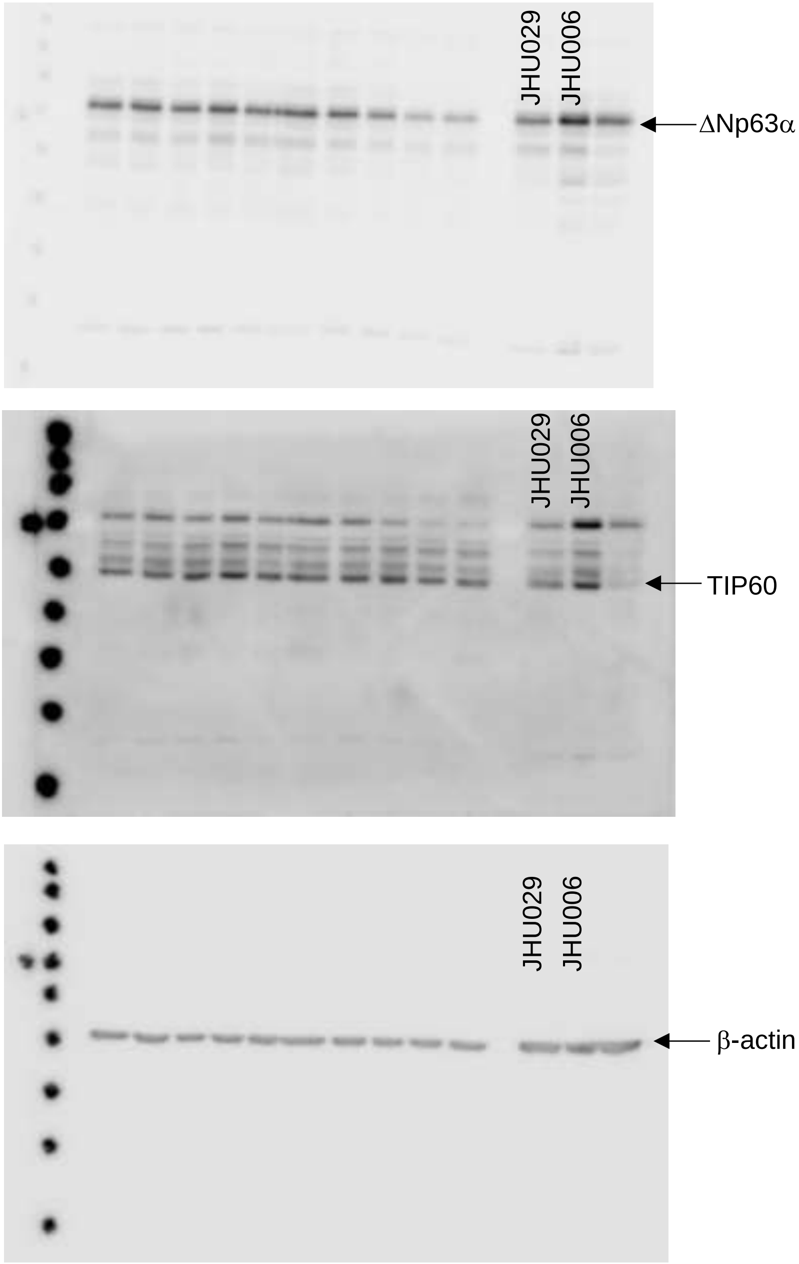

Figure-2A (JHU029 and JHU006)

IP-Acetyl K

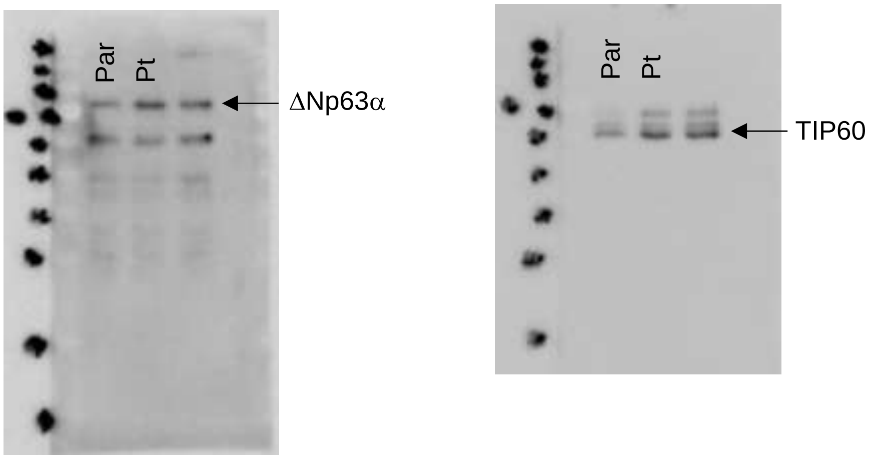

Input

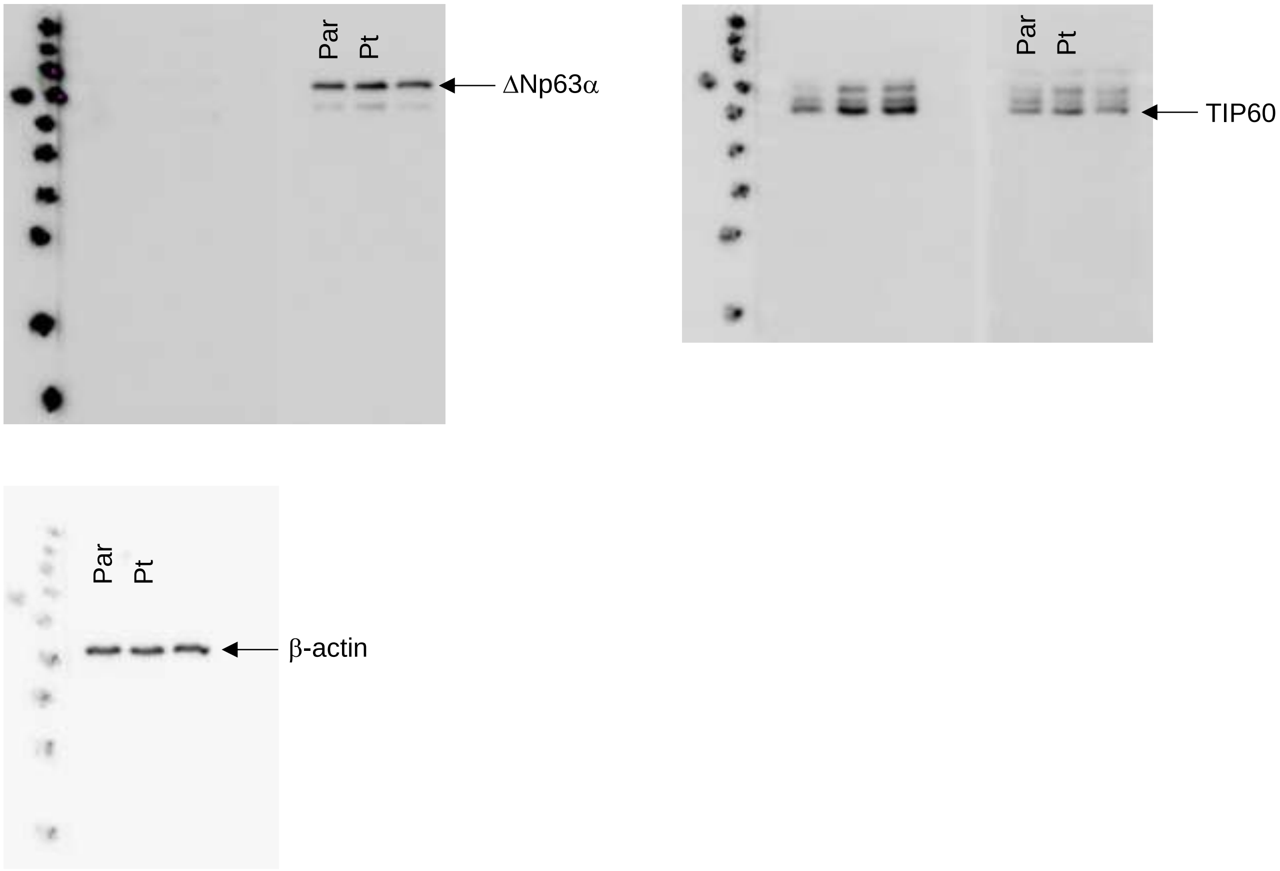

Figure-2B (A431 Pt)

IP-Acetyl K

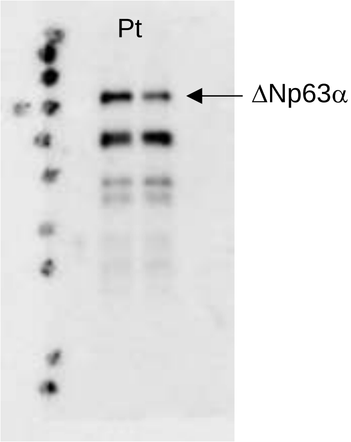

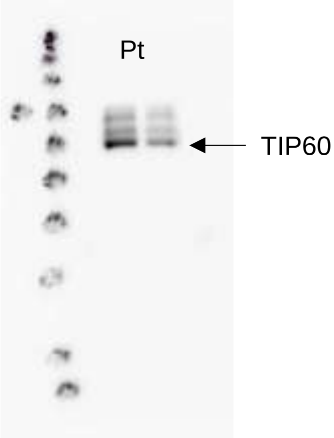

Input

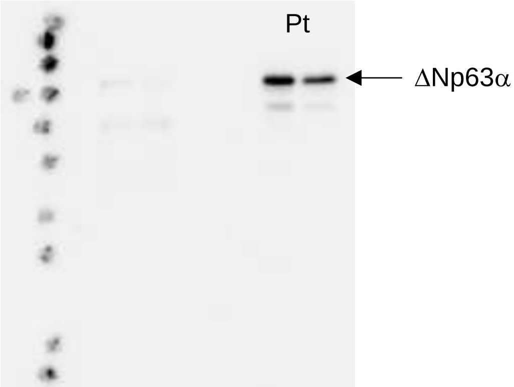

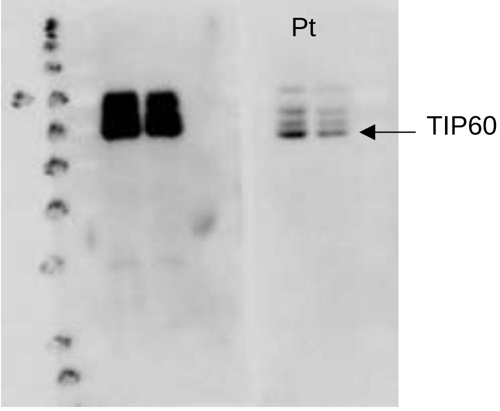

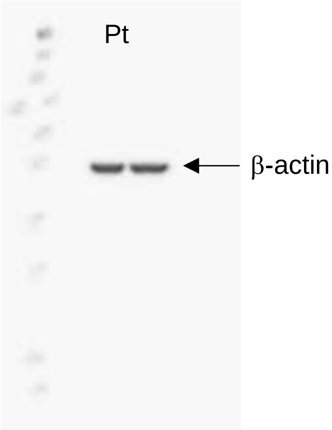

Figure-2B (JHU006)

IP-Acetyl K

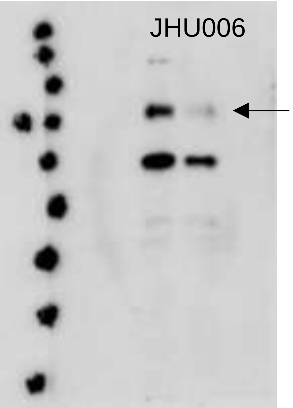

ΔNp63α

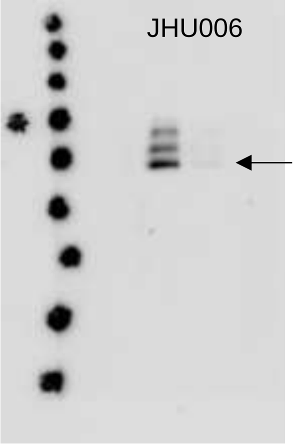

TIP60

Input

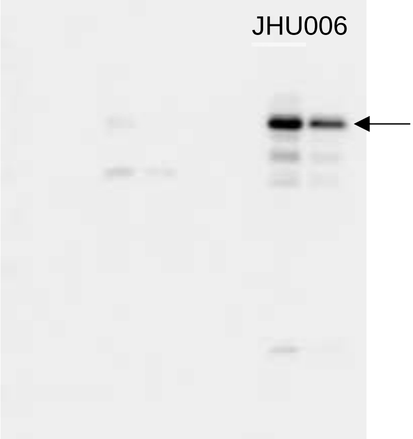

ΔNp63α

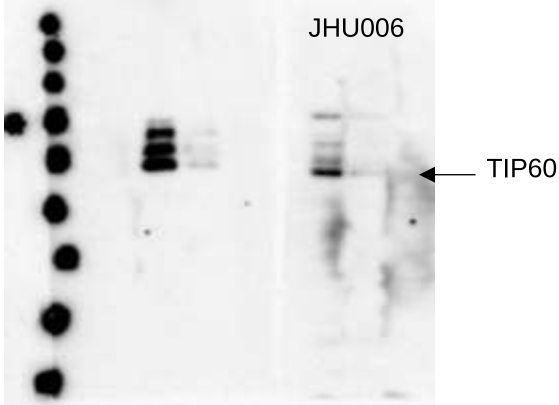

TIP60

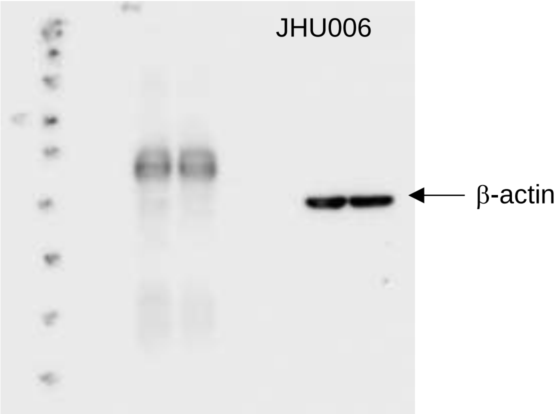

β-actin

Figure-2C (A431 Pt)

IP-Acetyl K

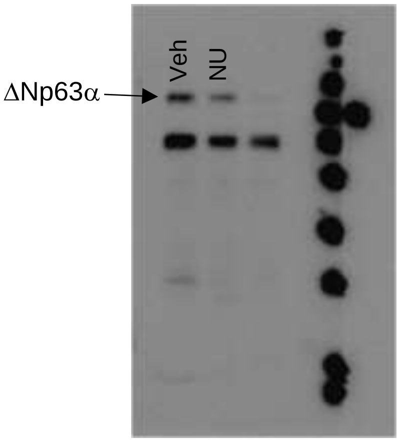

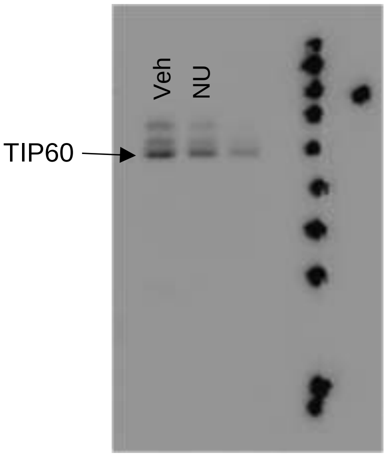

Input

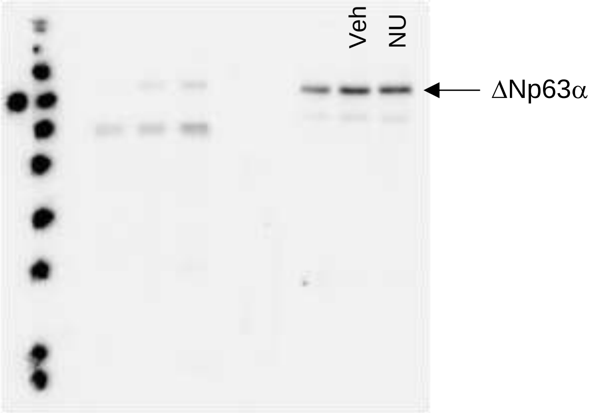

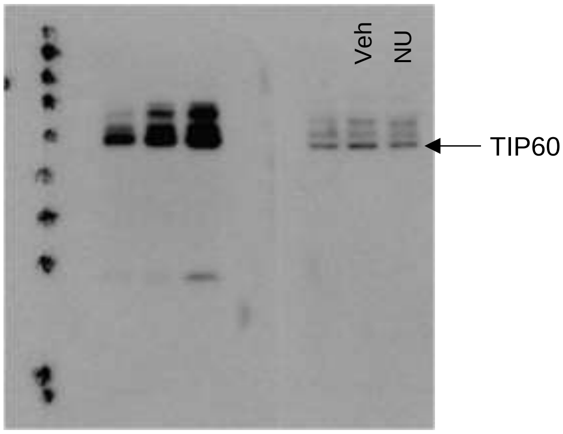

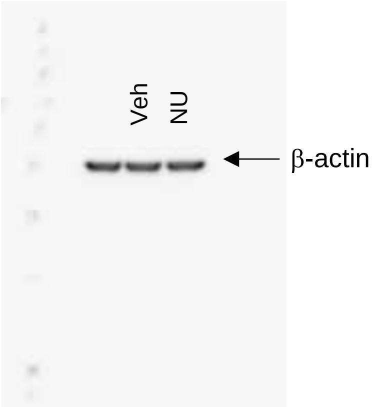

Figure-2C (JHU006)

IP-Acetyl K

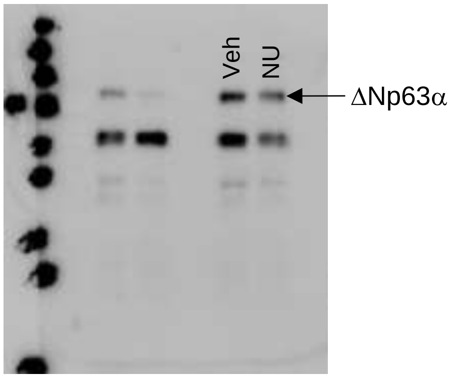

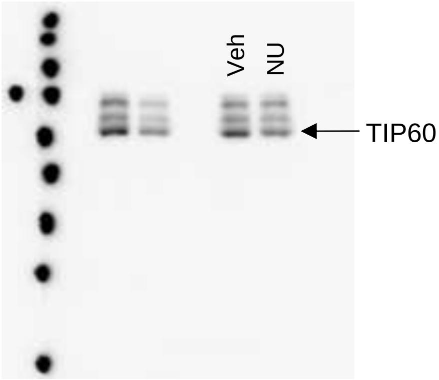

Input

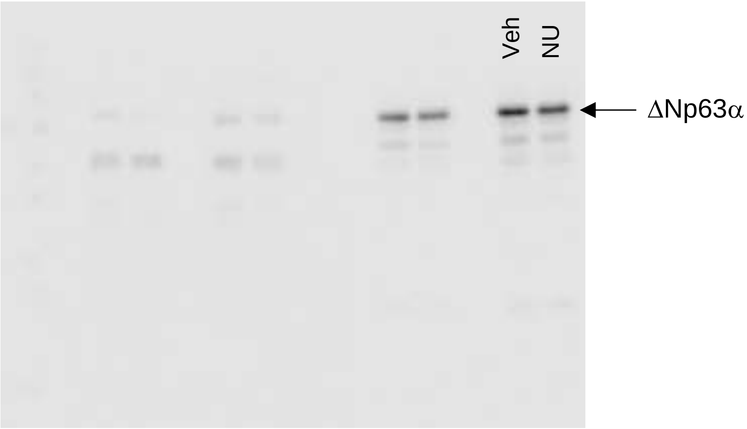

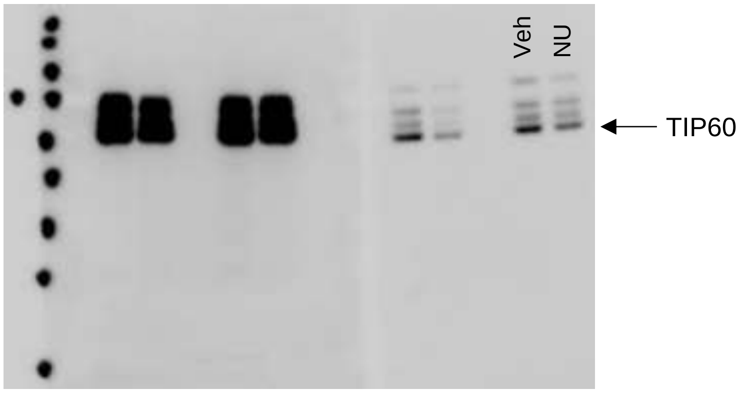

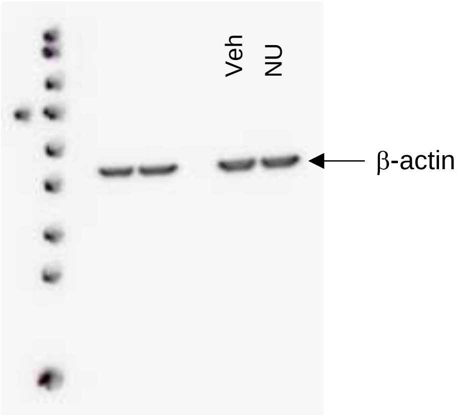

Figure-2D (A431 Parental and Pt)

IP-Acetyl K

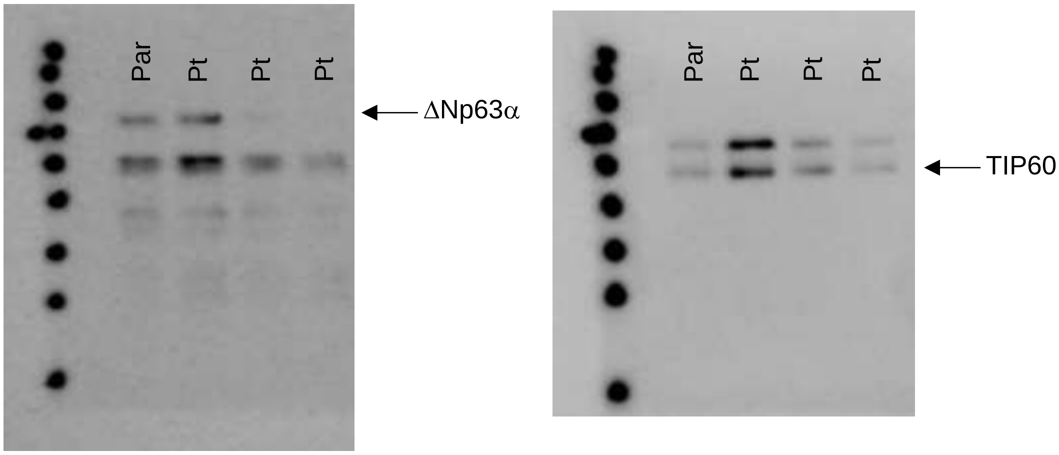

Input

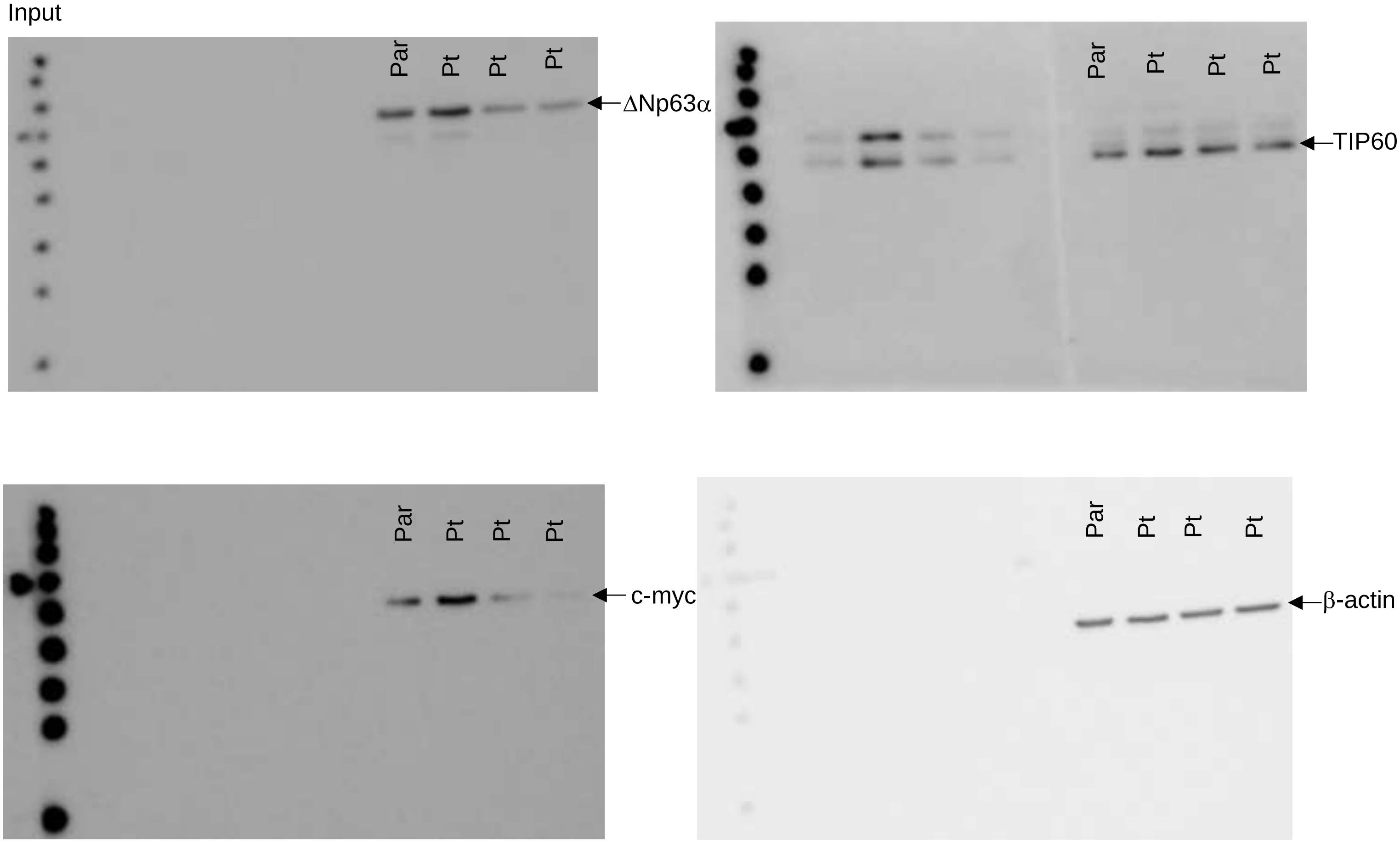

Figure-2D (JHU006)

IP-Acetyl K

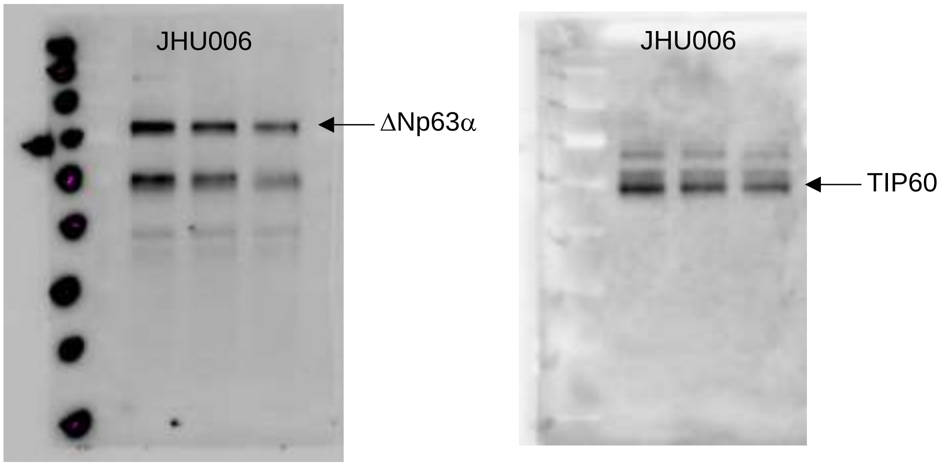

Input

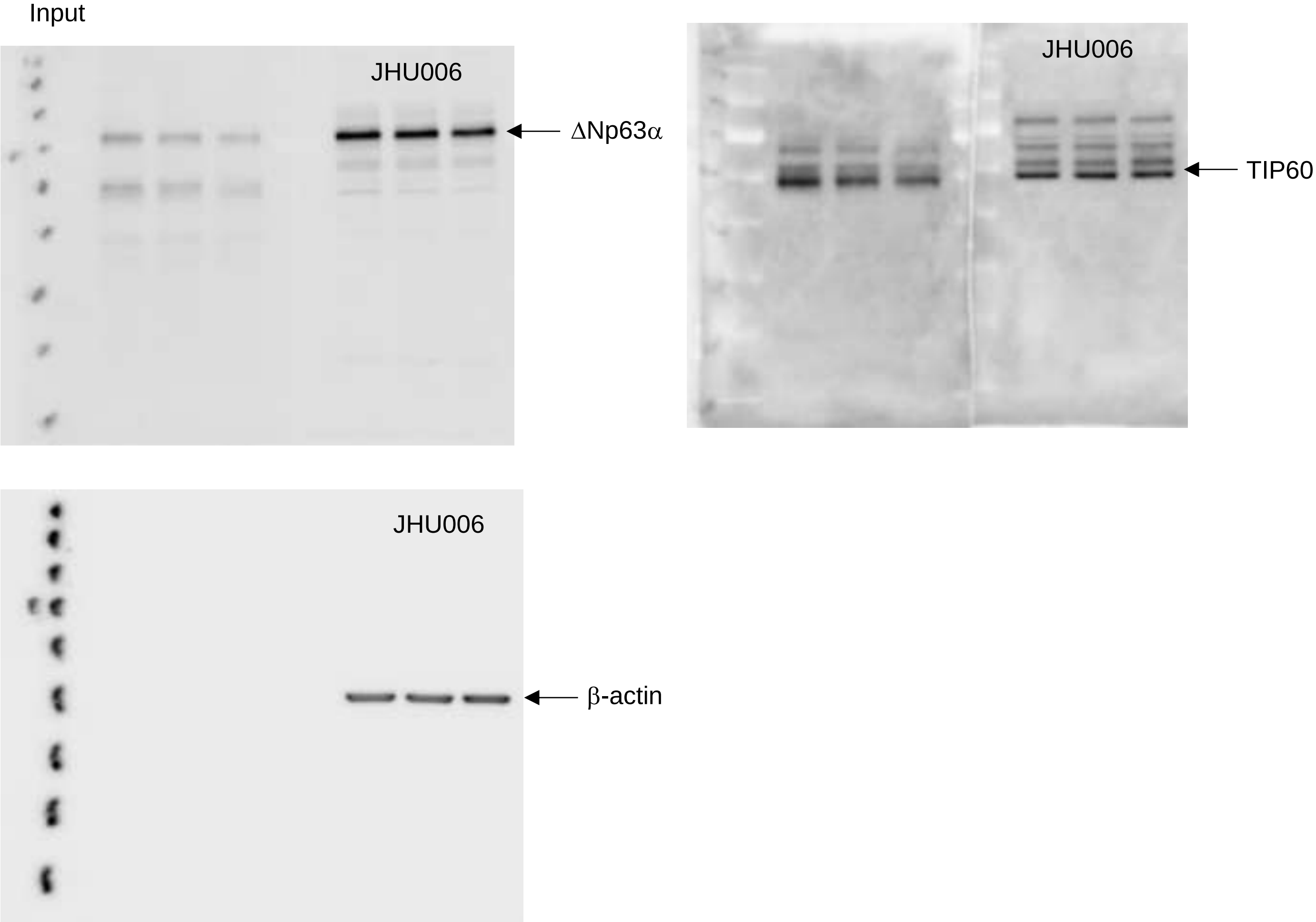

Figure-2E (A431 Parental and Pt)

IP-Acetyl K

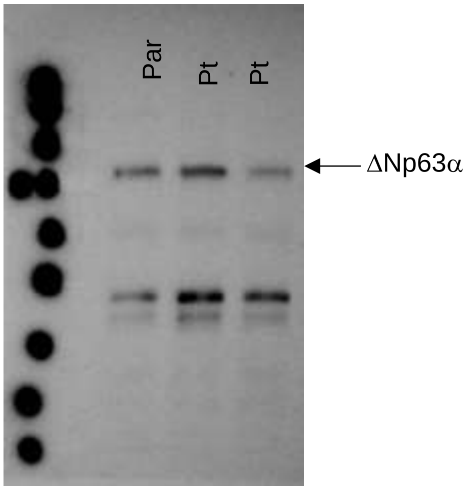

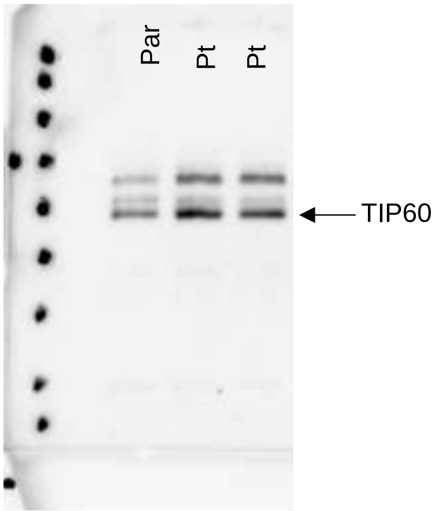

Input

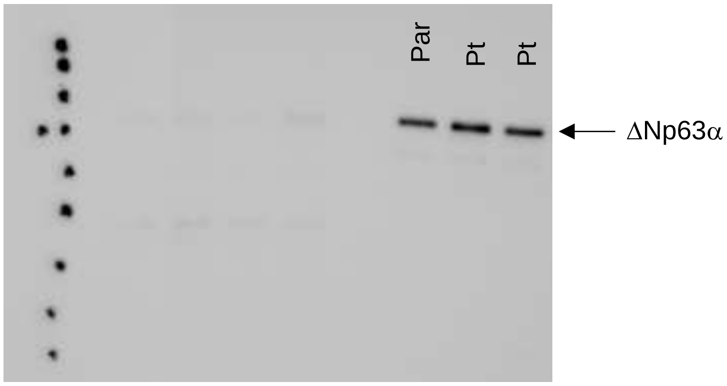

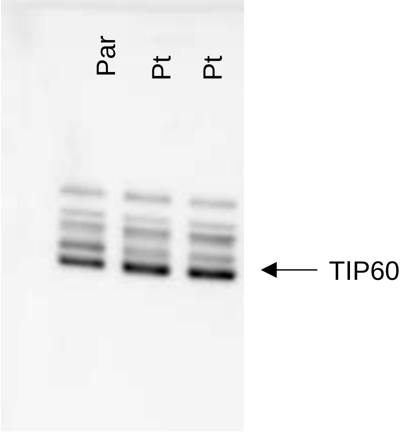

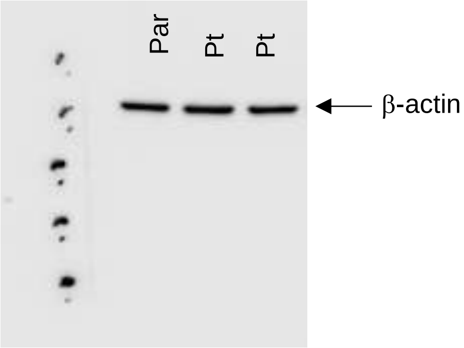

Figure-3A (A431 Parental and Pt)

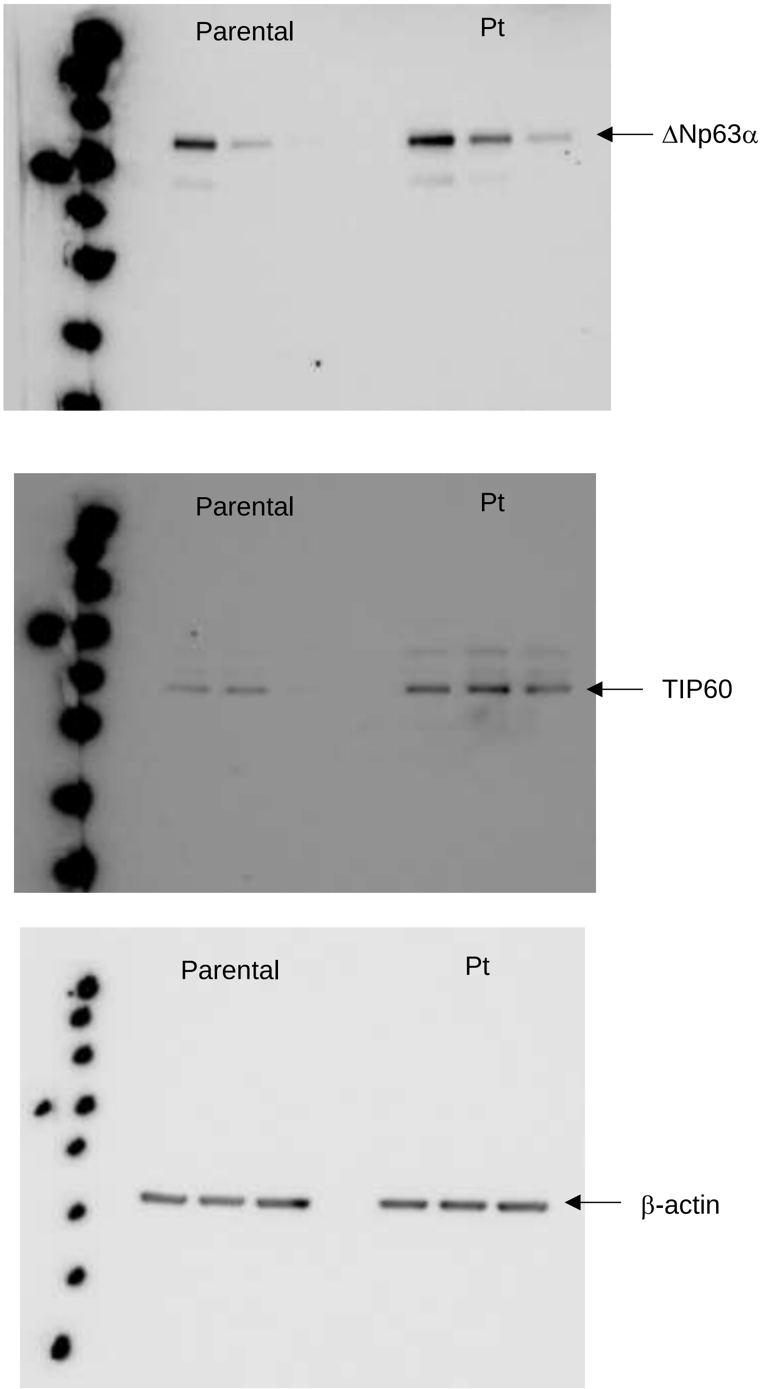

Figure-3B (A431 Parental and Pt)

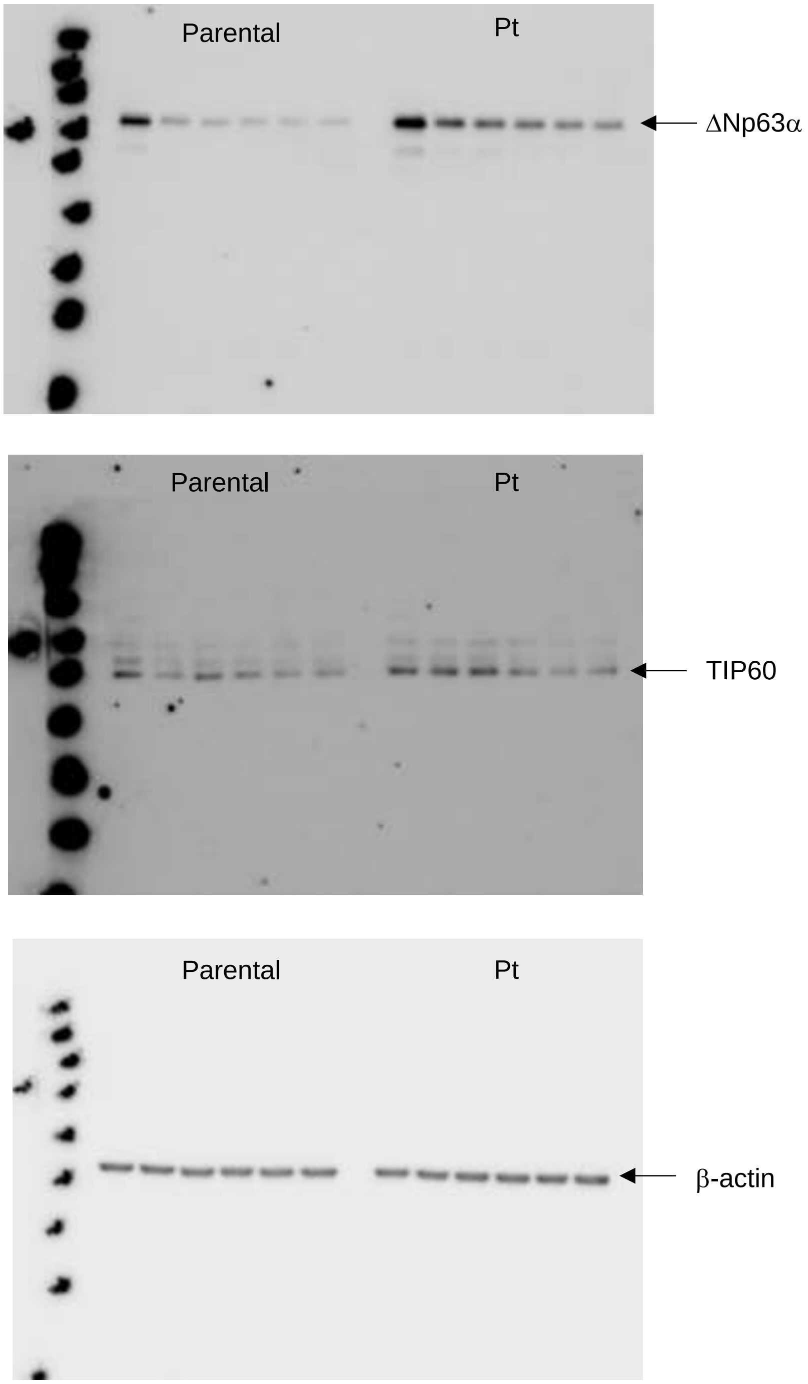

Figure-3C (Lenti-A431 eGFP and TIP60)

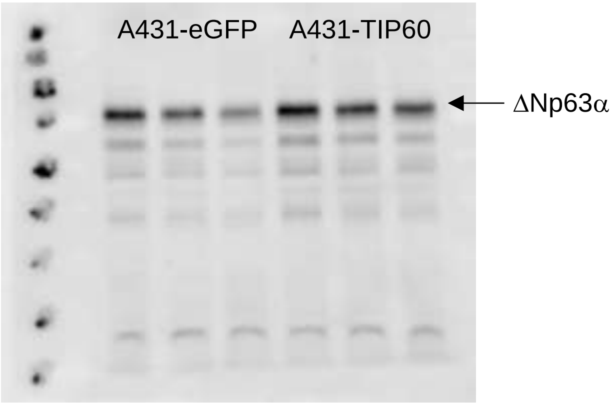

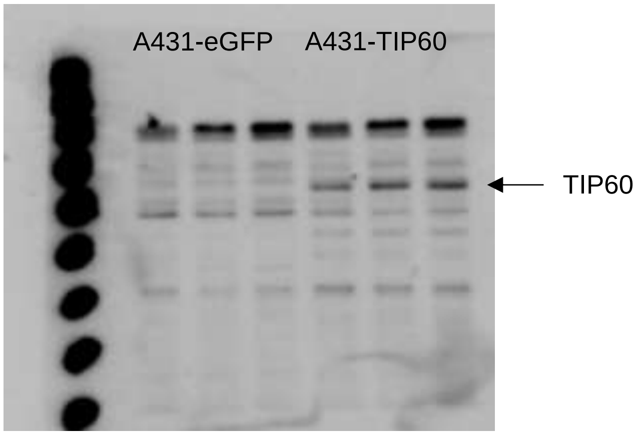

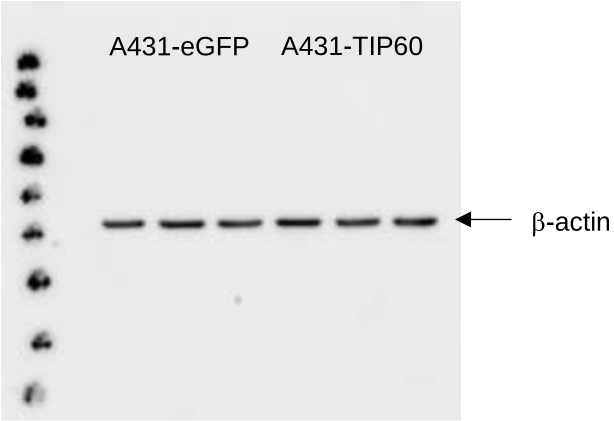

Figure-4A (A431 Pt)

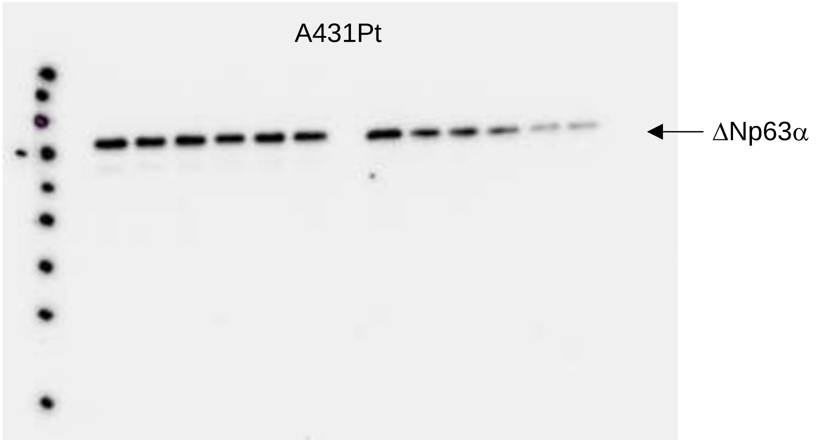

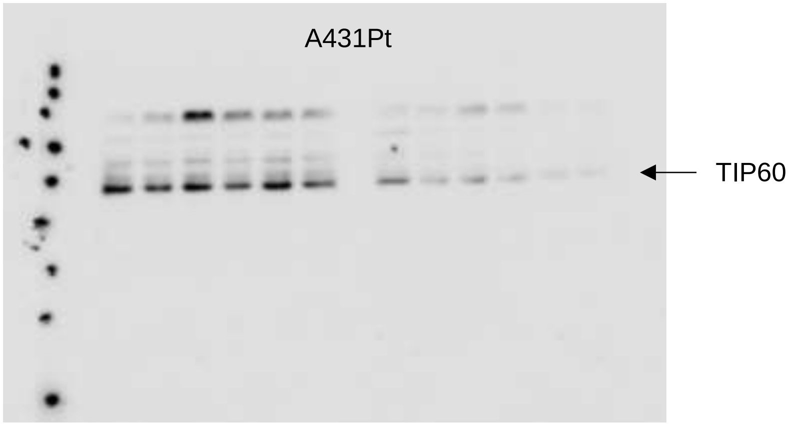

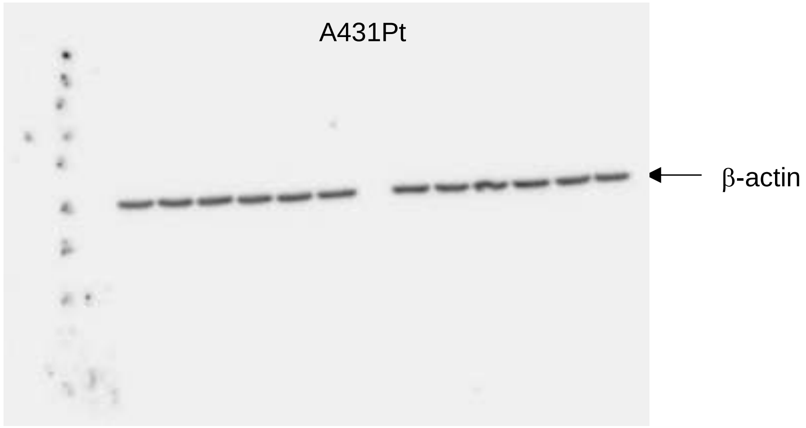

Figure-4B (A431 Pt)

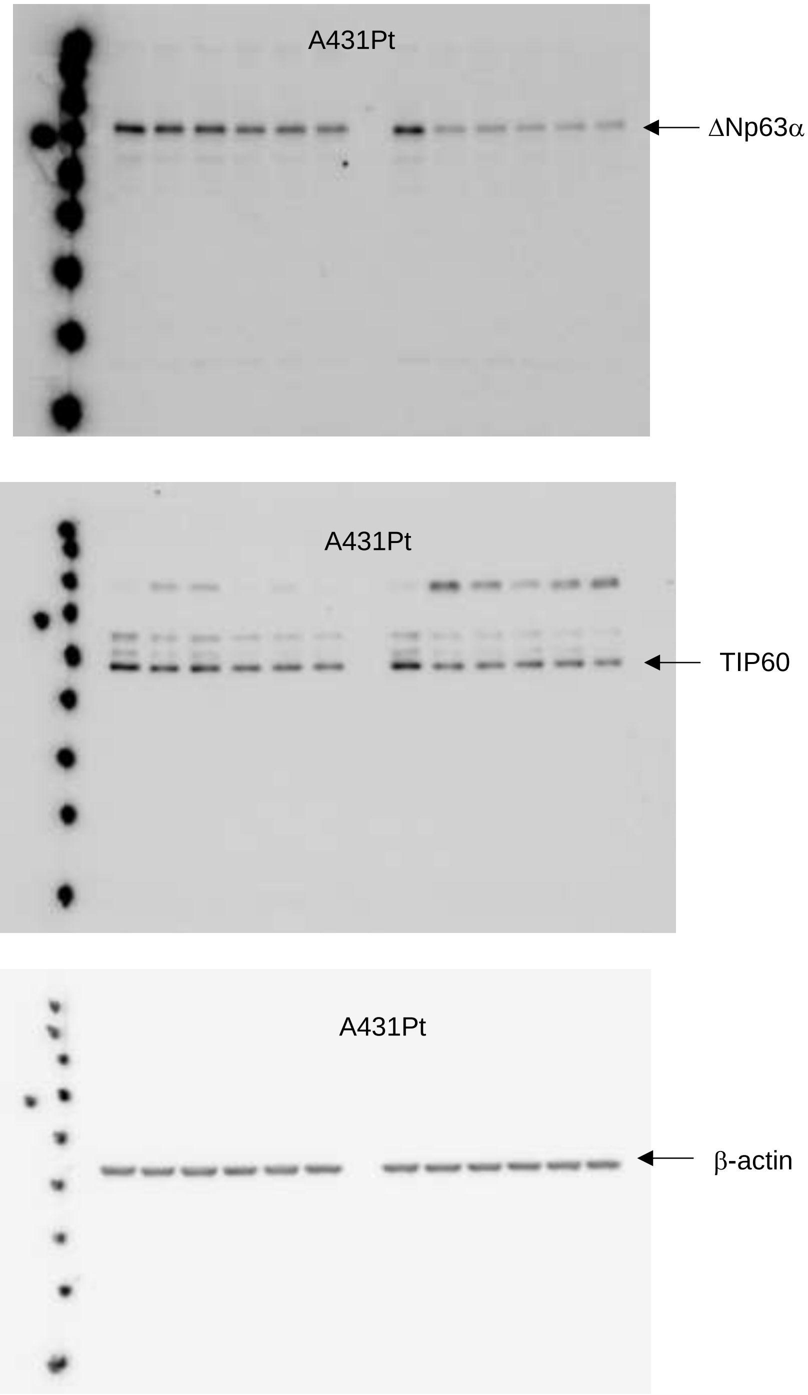

Figure-4C (JHU006)

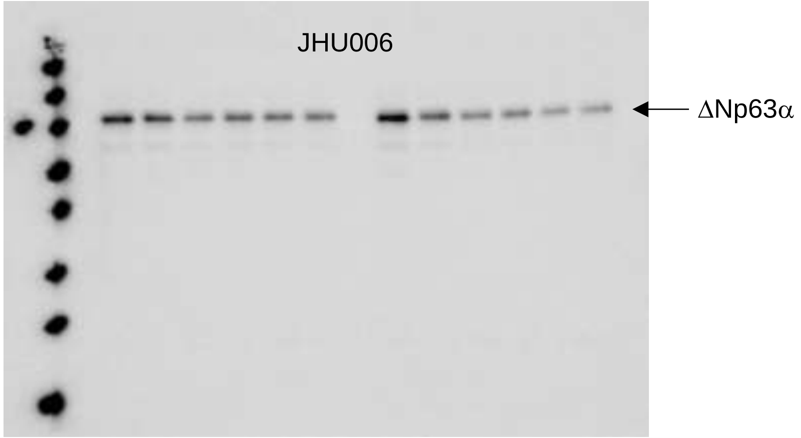

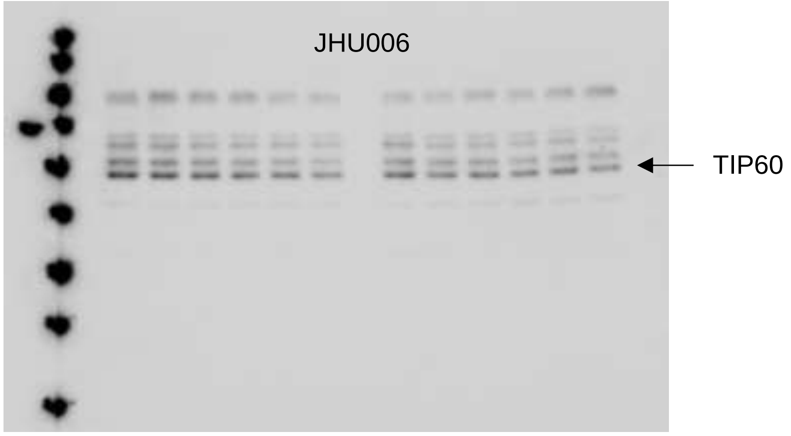

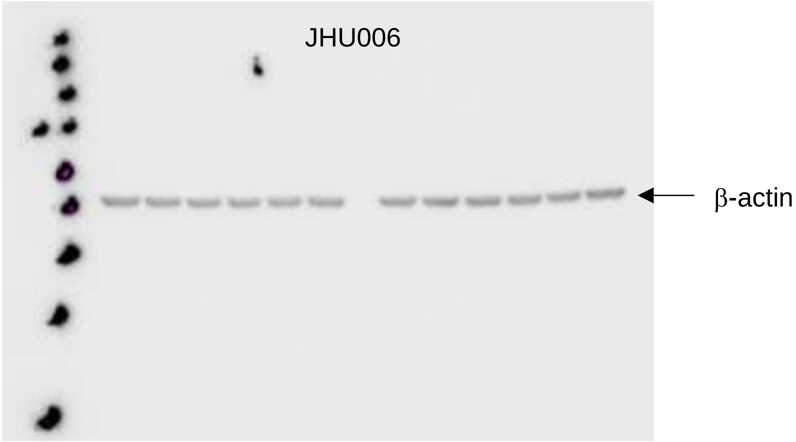

Figure-5A (A431 Parental and Pt)

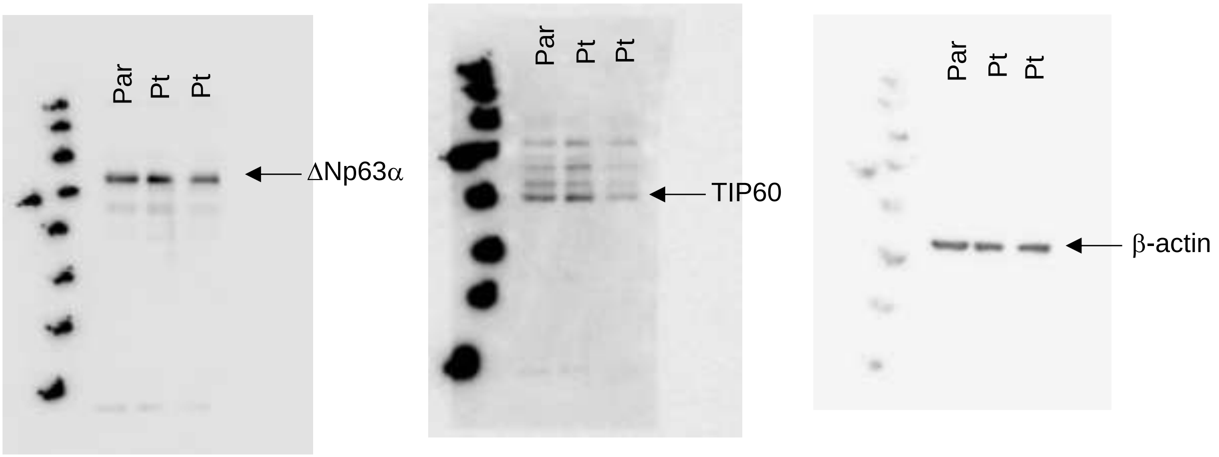

Figure-5B (JHU029 and JHU006)

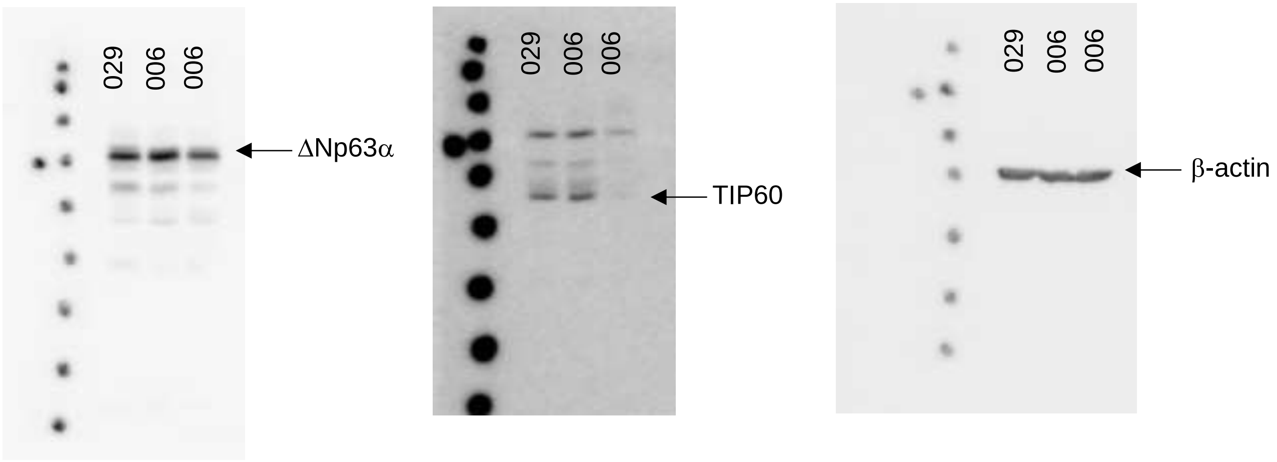

Figure-5C (A431 Parental and Pt NU)

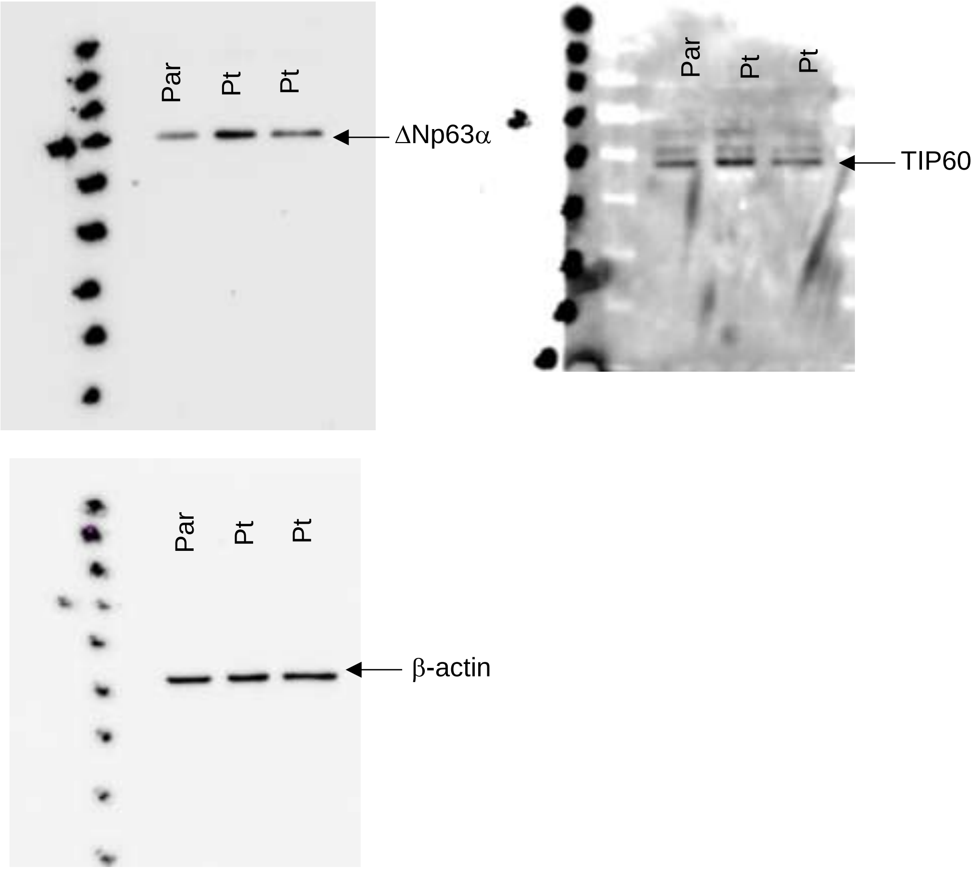

Figure-5D (A431 Parental and Pt TH)

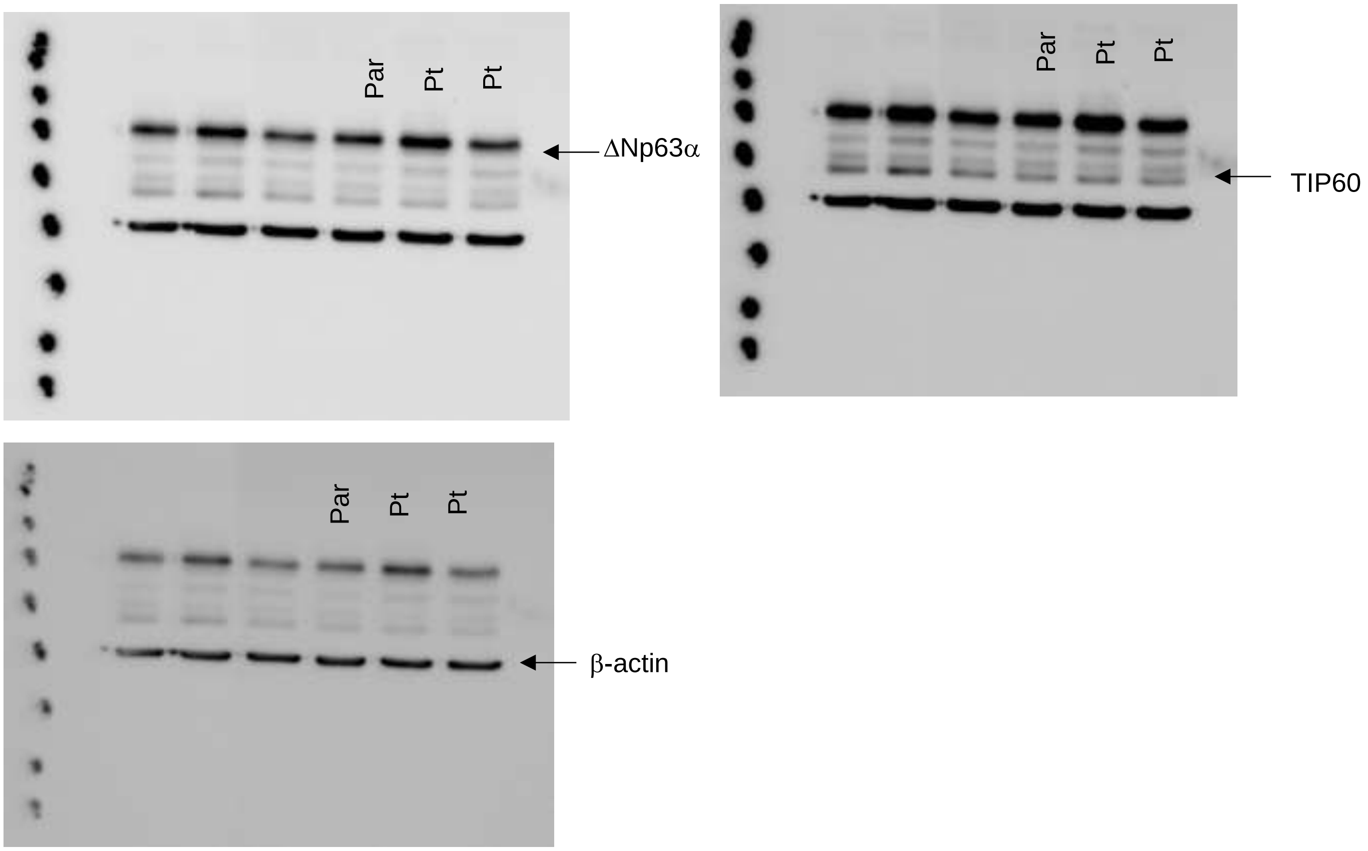

Figure-5E (Lenti-A431 eGFP, ΔNp63α and TIP60)

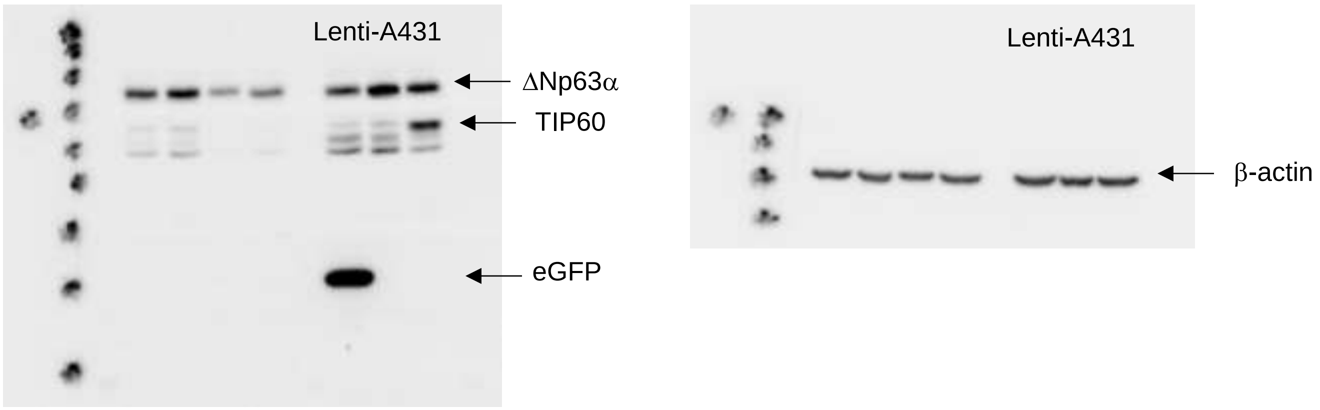

Figure-5F (Lenti-JHU029 eGFP, ΔNp63α and TIP60)

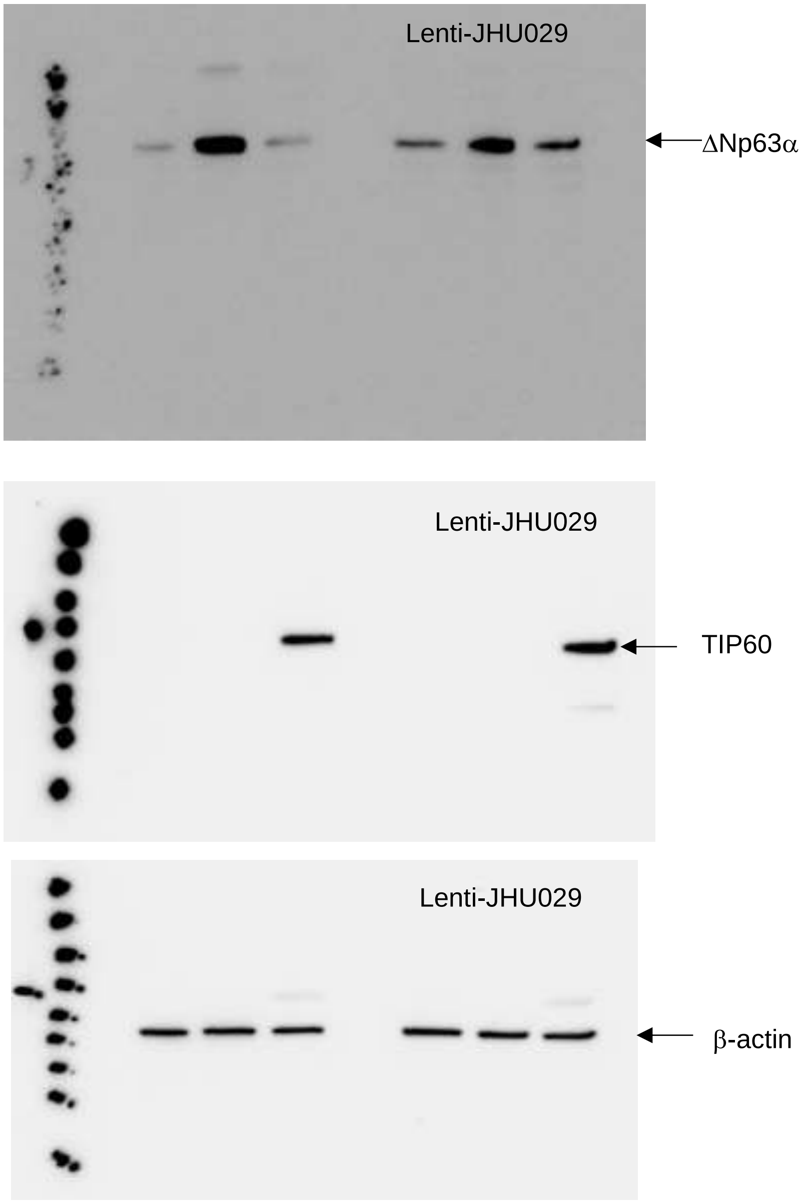

Figure-7B (A431 Parental and Pt G2/M)

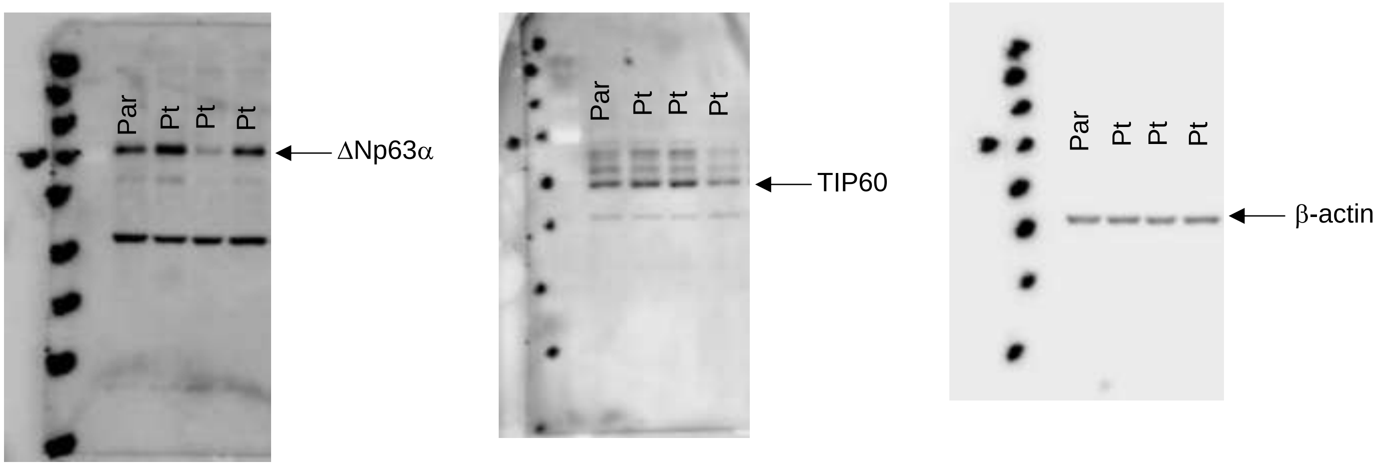

Figure-7C (A431 Pt si p21) and 7E (A431 Pt and JHU006 TH)

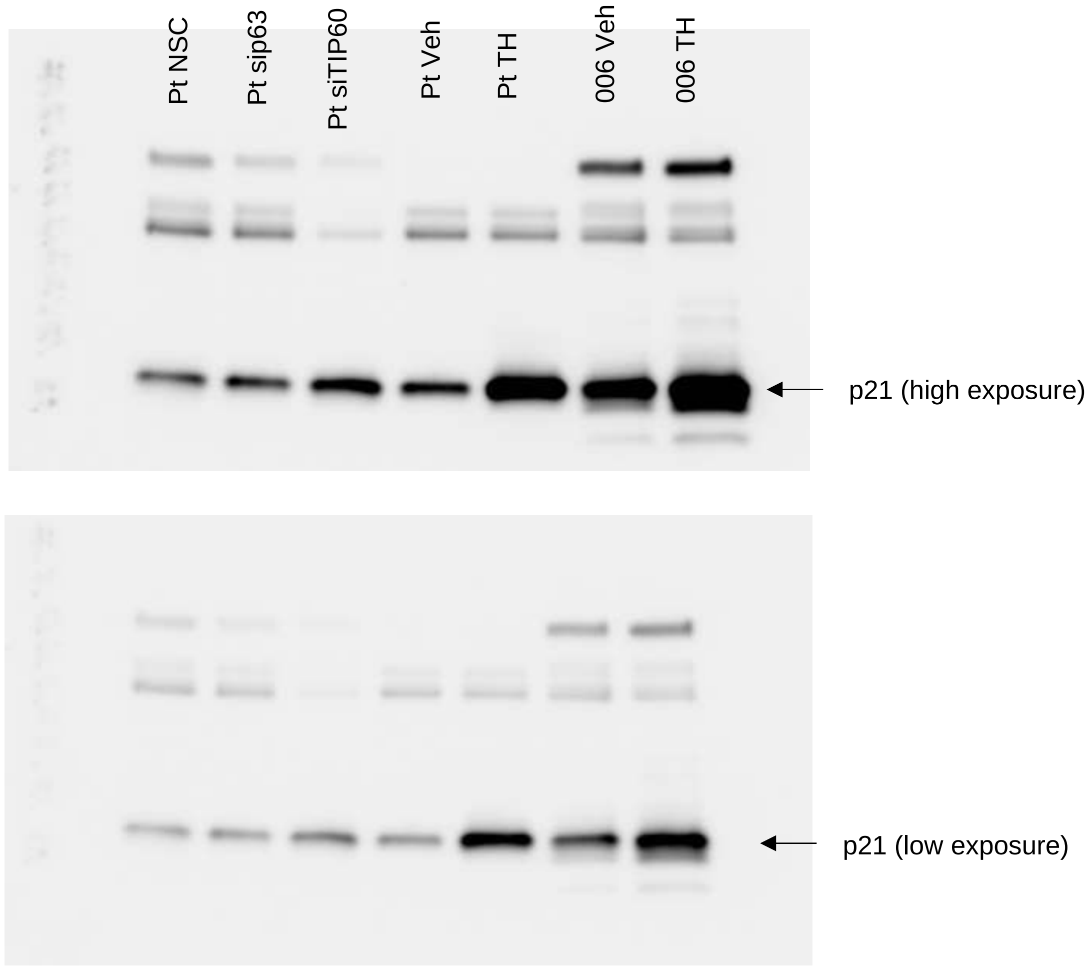

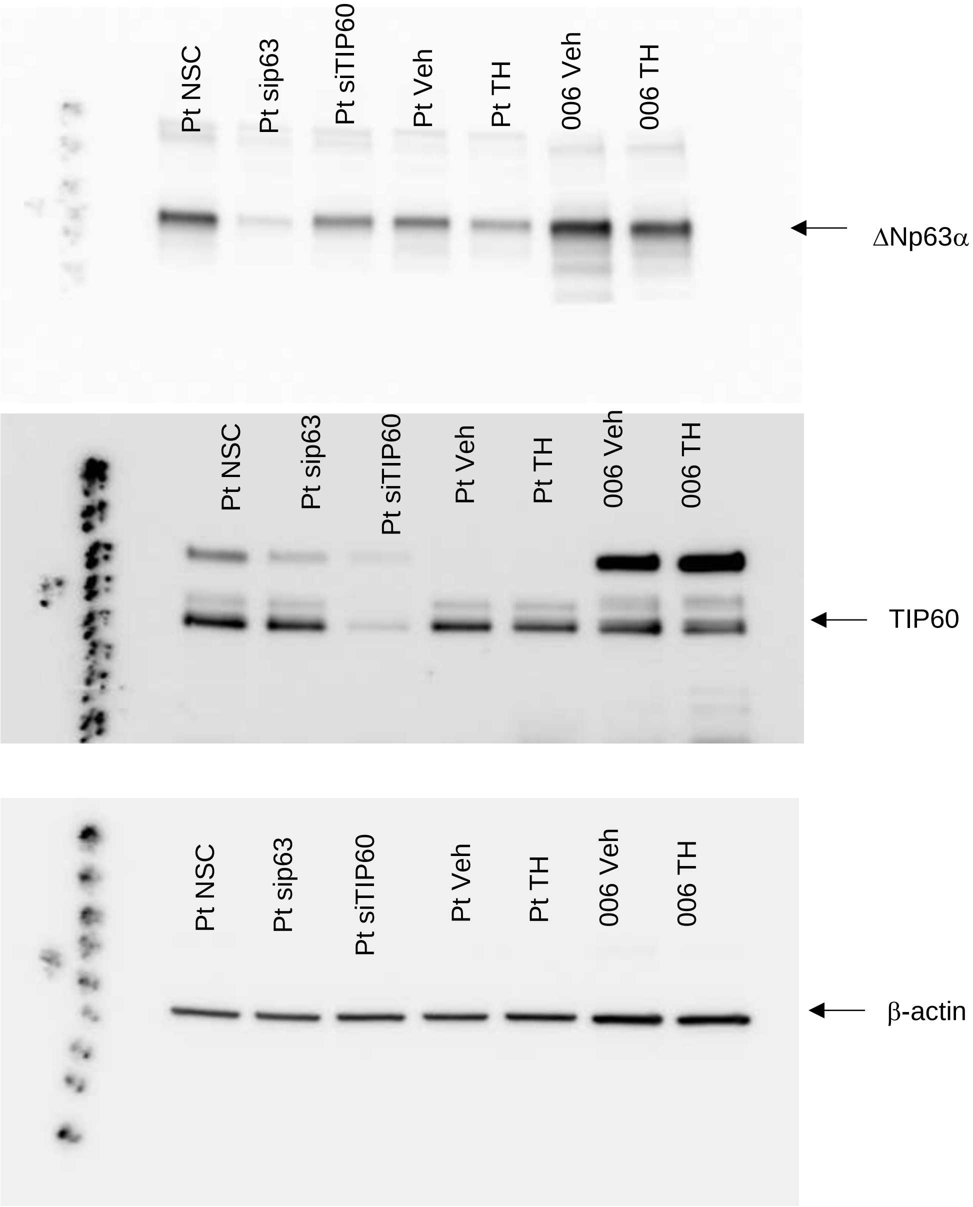
Pt NSC
Pt sip63
Pt siTIP60
Pt Veh
Pt TH
006 Veh
006 TH
ΔNp63α
Pt NSC
Pt sip63
Pt siTIP60
Pt Veh
Pt TH
006 Veh
006 TH
TIP60
Pt NSC
Pt sip63
Pt siTIP60
Pt Veh
Pt TH
006 Veh
006 TH
β-actin

Figure-8B (A431 Parental and Pt Sub-G1)

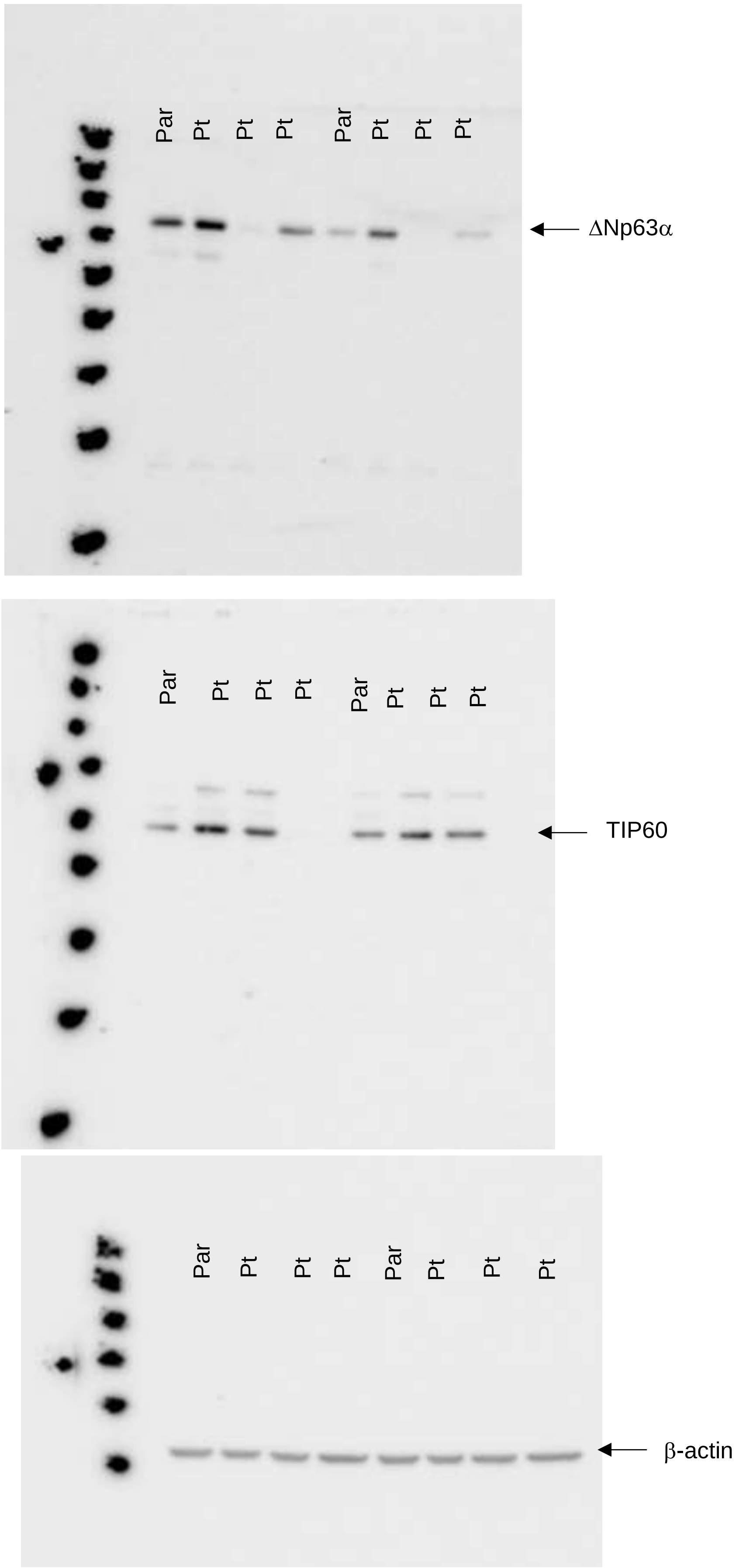

Figure-8C (A431 Parental and Pt si)

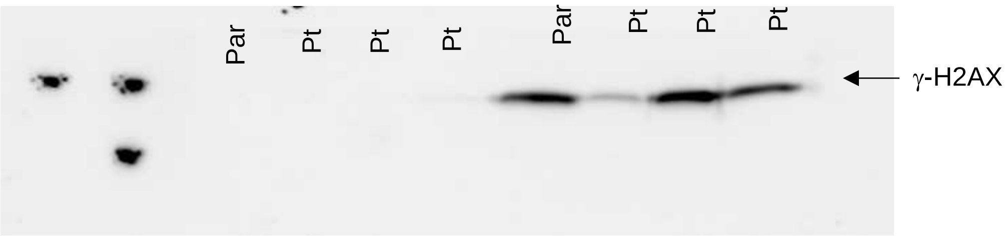

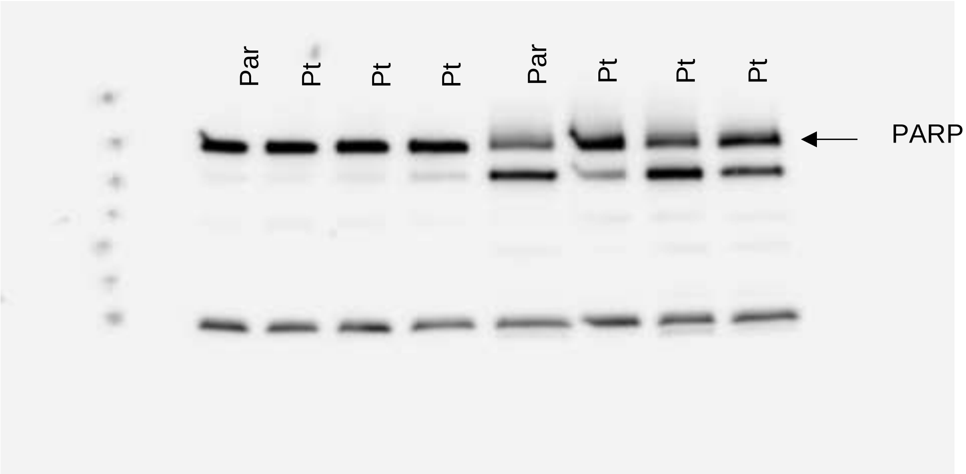

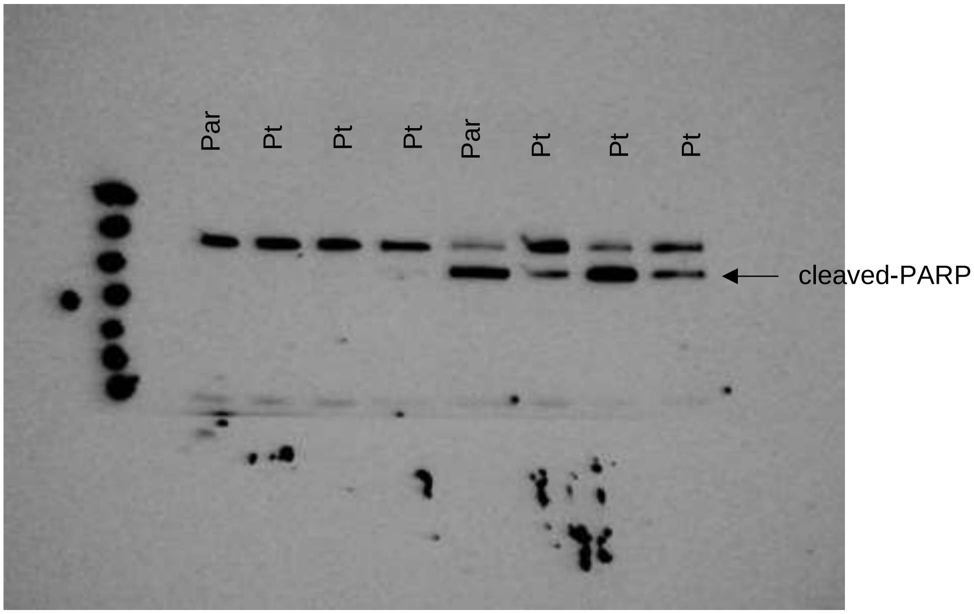

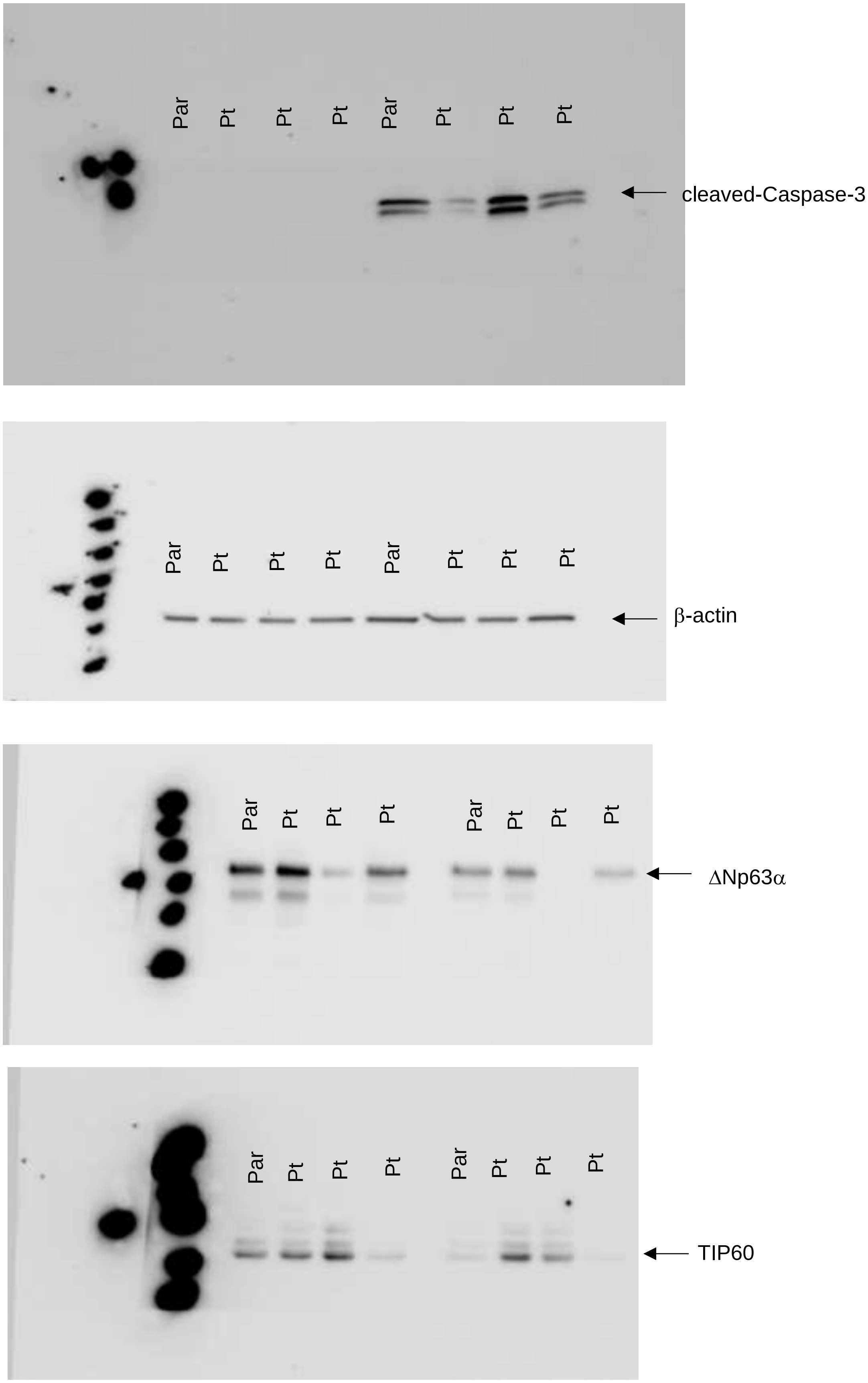

Par Pt Pt Pt Par Pt Pt Pt
cleaved-Caspase-3
Par Pt Pt Pt Par Pt Pt Pt
β-actin
Par Pt Pt Pt Par Pt Pt Pt
ΔNp63α
Par Pt Pt Pt Par Pt Pt Pt
TIP60

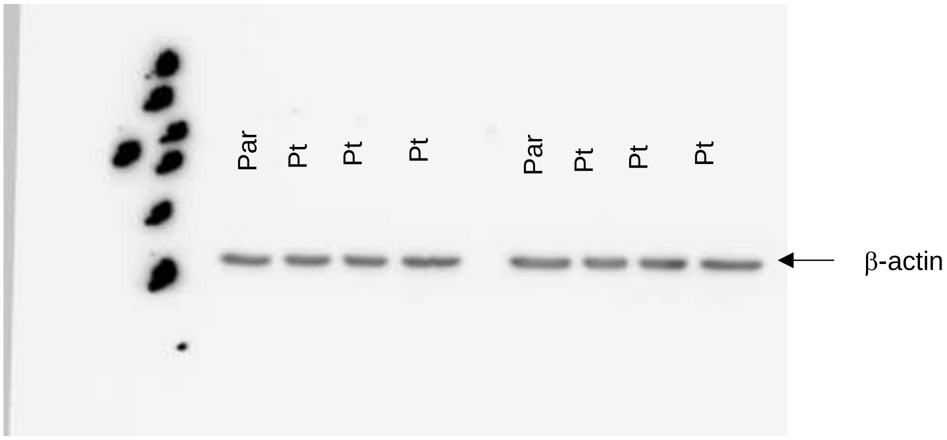
Par
Pt
Pt
Pt
Par
Pt
Pt
Pt
β-actin

Figure-8D (A431 Parental and Pt TH)

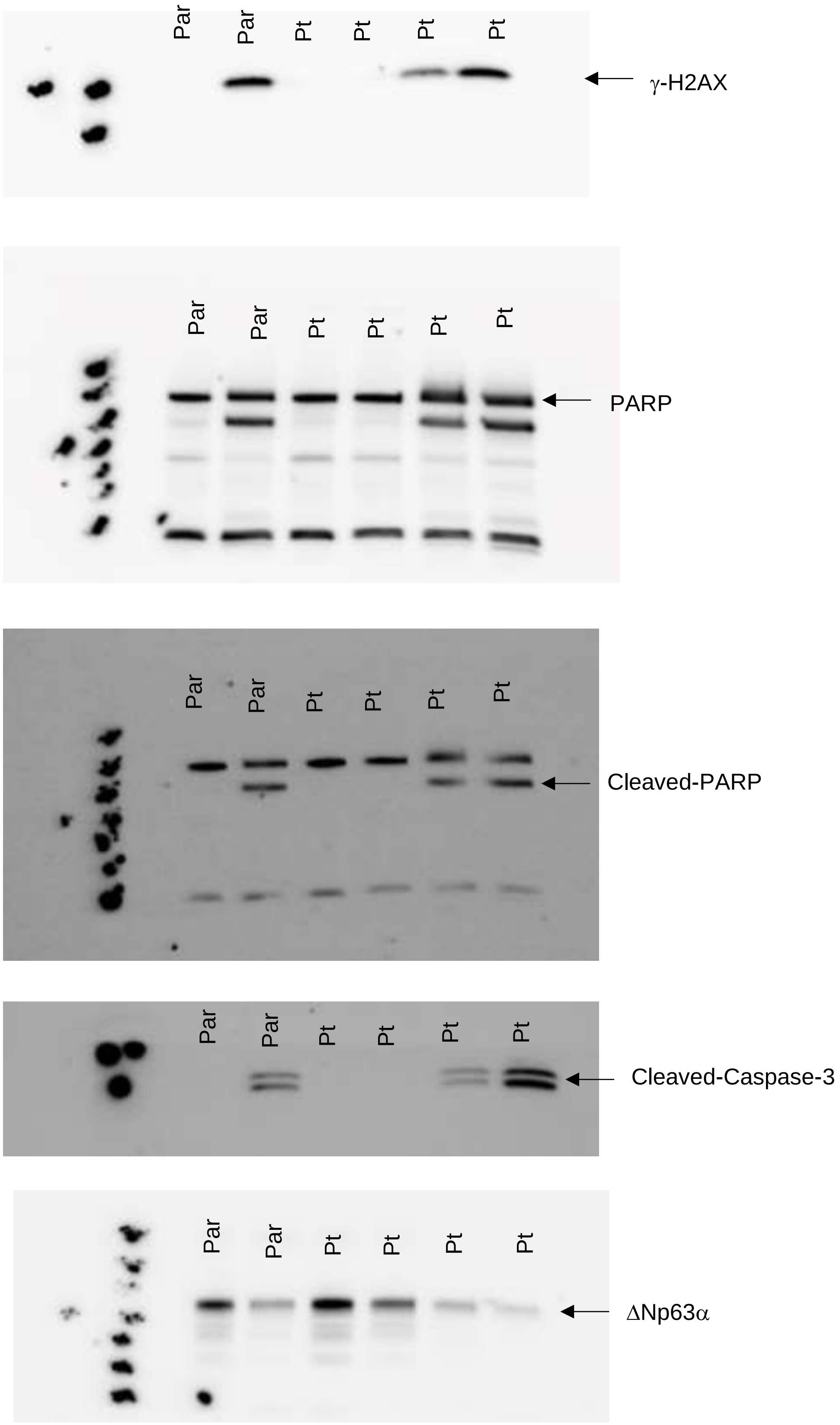

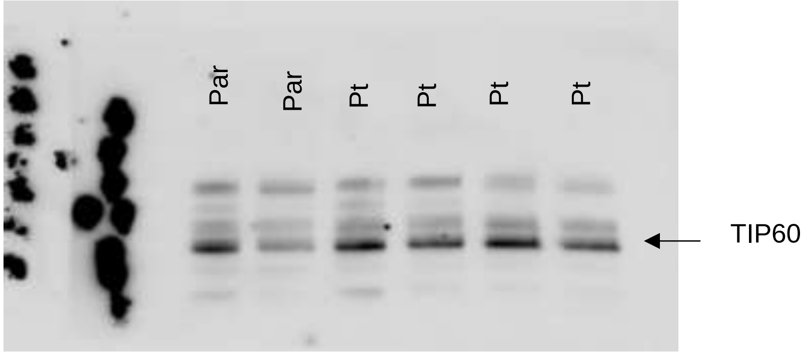
Par
Par
Pt
Pt
Pt
Pt
TIP60

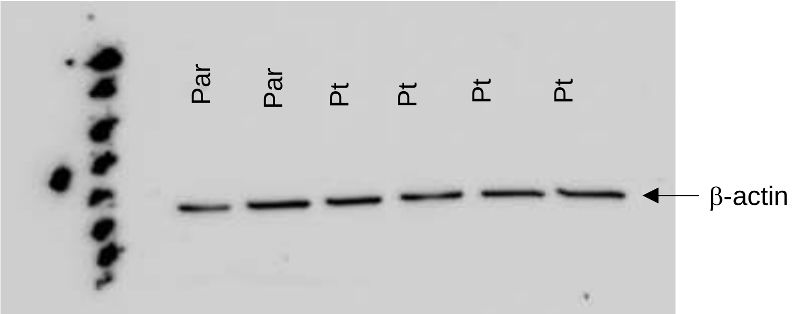
Par
Par
Pt
Pt
Pt
Pt
β-actin

Supplementary Figure-2A (JHU006 sh ctrl and shTIP60)

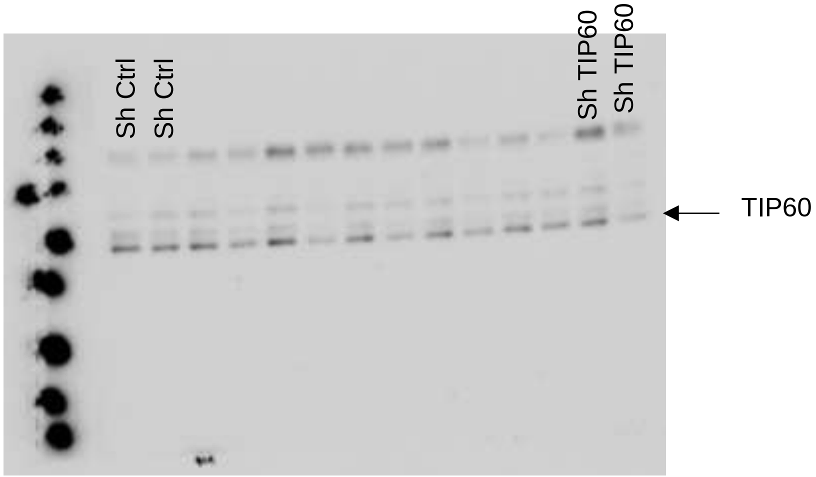

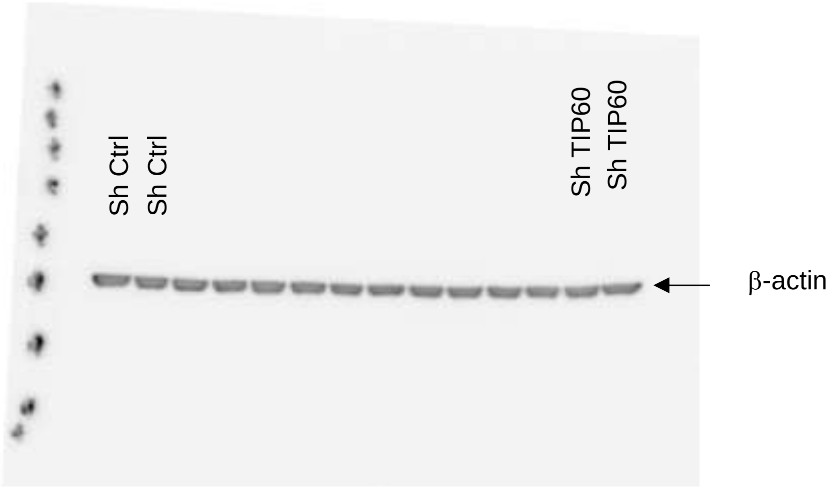

Supplementary Figure-2B (JHU006 sh ctrl and shTIP60)

IP-Acetyl K

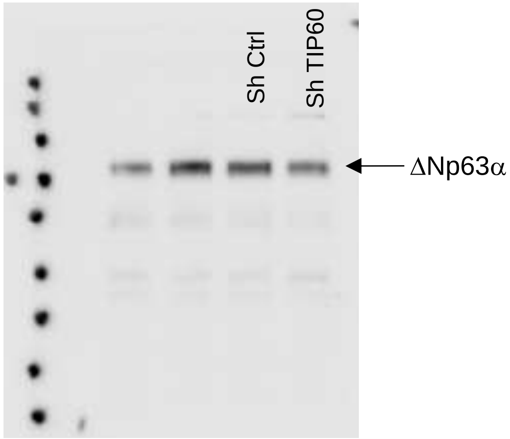

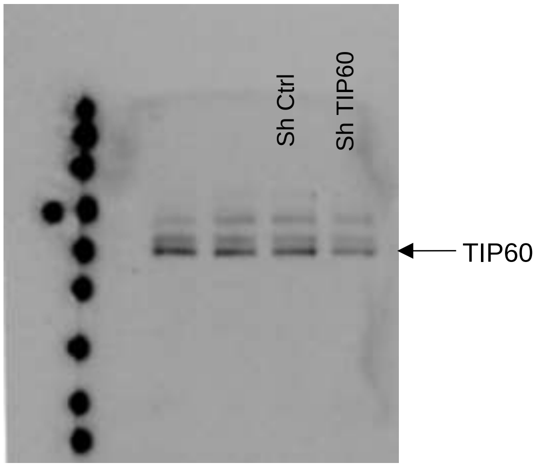

Input

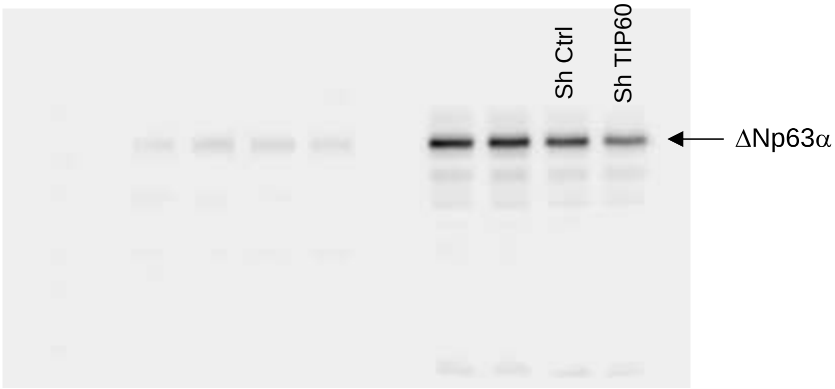

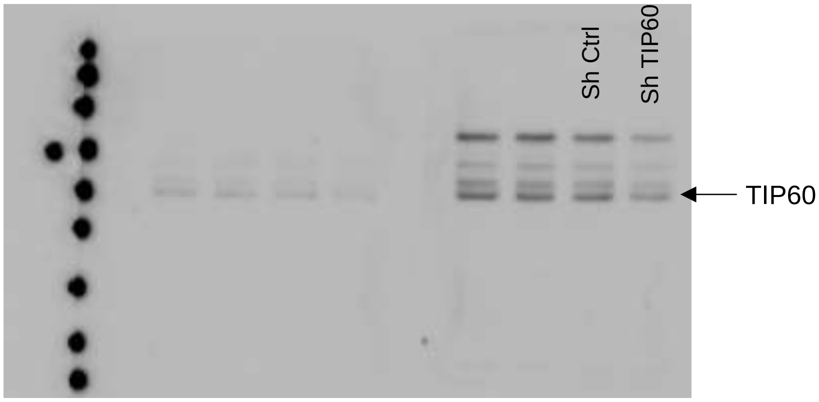

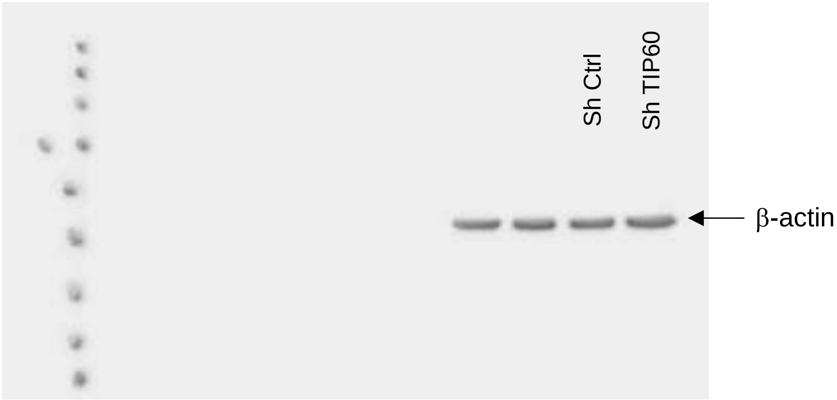

Supplementary Figure-4C (Lenti-A431 eGFP and ΔNp63α)

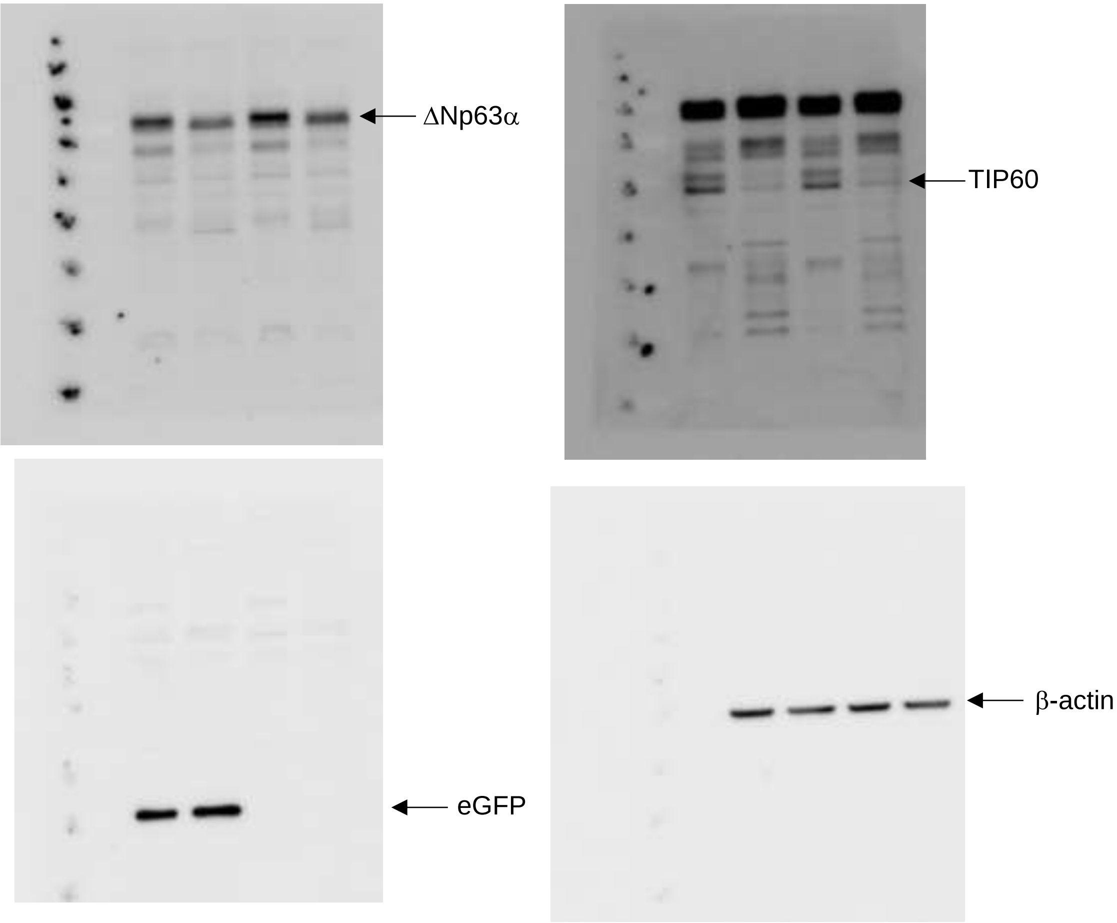

Supplement: Supplementary file 2 — Supplemental material Westrn blot images [file 41419_2024_7265_MOESM2_ESM.pdf]
